# Chromosome-Scale Assembly of the *Dendrobium nobile* Genome Provides Insights Into the Molecular Mechanism of the Biosynthesis of the Medicinal Active Ingredient of *Dendrobium*

**DOI:** 10.3389/fgene.2022.844622

**Published:** 2022-03-01

**Authors:** Qing Xu, Shan-Ce Niu, Kang-Li Li, Pei-Ji Zheng, Xiao-Jing Zhang, Yin Jia, Yang Liu, Yun-Xia Niu, Li-Hong Yu, Duan-Fen Chen, Guo-Qiang Zhang

**Affiliations:** ^1^ GMU-GIBH Joint School of Life Sciences, Guangzhou Medical University, Guangzhou, China; ^2^ College of Horticulture, Hebei Agricultural University, Baoding, China; ^3^ State Key Laboratory of North China Crop Improvement and Regulation, Hebei Agricultural University, Baoding, China; ^4^ School of Vocational Education, Tianjin University of Technology and Education, Tianjin, China; ^5^ Laboratory for Orchid Conservation and Utilization, The Orchid Conservation and Research Center of Shenzhen, The National Orchid Conservation Center of China, Shenzhen, China

**Keywords:** *Dendrobium nobile*, chromosome-level assembly, polysaccharide and alkaloid (dendrobine), gene family, transcriptome

## Abstract

Orchids constitute approximately 10% of flowering plant species. However, only about 10 orchid genomes have been published. Metabolites are the main way through which orchids respond to their environment. *Dendrobium nobile*, belonging to *Dendrobium*, the second largest genus in Orchidaceae, has high ornamental, medicinal, and ecological value. *D. nobile* is the source of many popular horticultural varieties. Among the *Dendrobium* species, *D. nobile* has the highest amount of dendrobine, which is regarded as one of the criteria for evaluating medicinal quality. Due to lack of data and analysis at the genomic level, the biosynthesis pathways of dendrobine and other related medicinal ingredients in *D. nobile* are unknown. In this paper, we report a chromosome-scale reference genome of *D. nobile* to facilitate the investigation of its genomic characteristics for comparison with other *Dendrobium* species. The assembled genome size of *D. nobile* was 1.19 Gb. Of the sequences, 99.45% were anchored to 19 chromosomes. Furthermore, we identified differences in gene number and gene expression patterns compared with two other *Dendrobium* species by integrating whole-genome sequencing and transcriptomic analysis [e.g., genes in the polysaccharide biosynthesis pathway and upstream of the alkaloid (dendrobine) biosynthesis pathway]. Differences in the *TPS* and *CYP450* gene families were also found among orchid species. All the above differences might contribute to the species-specific medicinal ingredient biosynthesis pathways. The metabolic pathway-related analysis will provide further insight into orchid responses to the environment. Additionally, the reference genome will provide important insights for further molecular elucidation of the medicinal active ingredients of *Dendrobium* and enhance the understanding of orchid evolution.

## Introduction

Jinchai Shihu (金钗石斛) *Dendrobium nobile* is a medicinal species belonging to *Dendrobium*, the second largest genus in the Orchidaceae. With high amounts of medicinal active ingredients, *D. nobile* is also one of the five *Dendrobium* species recorded in the Chinese Pharmacopoeia (2020 Edition). Polysaccharides and alkaloids are the main medicinal components of *Dendrobium*. They are mainly stored in the stem and have strong antioxidant, neuroprotective, antidiabetic, antihypertensive, and immunomodulatory activities (Wang et al., 2010; Li et al., 2011; Liu et al., 2011; Wang et al*.*, 2020). Dendrobine, a sesquiterpenoid alkaloid, is the signature bioactive component and is regarded as one of the criteria for evaluating the quality of *D. nobile*. The biosynthesis of these medicinal ingredients varies with factors including the tissues sampled, species, and genetics (Li et al., 2019). Elucidating the biosynthesis pathway of polysaccharides and alkaloids in *Dendrobium* is an important research topic.

To date, many genes related to the biosynthesis of polysaccharides and alkaloids have been reported. *Dendrobium* contains active polysaccharides (Wang et al., 2014), with three main kinds in stems depending on the content; namely, non-starch mannan polysaccharides, glucose, and galactose (Ng et al., 2012). Thus far, many carbohydrate-related genes have been reported, such as genes encoding polysaccharide-metabolism-related enzymes involved in the biosynthesis of polysaccharides, monosaccharide-related genes, and the basic building blocks for polysaccharide-synthesis-related genes, as well as genes playing important roles in the regulatory mechanism of polysaccharide synthesis (Ren et al., 2020; Wang et al., 2020). The main kind of alkaloid in *Dendrobium* is dendrobine, a sesquiterpenoid alkaloid (Li et al., 2019). There are three main pathways involved in the upstream of alkaloid biosynthesis: the shikimate pathway, the methylerythritol phosphate (MEP) pathway, and the mevolonate (MVA) pathway; some genes involved in these three pathways have been reported, such as 3-dehydroquinate synthase, 1-deoxy-D-xylulose 5-phosphate synthase and 3-hydroxy-3-methyl-glutarylcoenzyme A reductase (Tzin et al., 2012; Wang et al., 2020). In addition, members of the terpene synthases (TPSs) and cytochrome P450 monooxygenases (CYP450s) have been reported to play an important role in dendrobine biosynthesis (Guo et al., 2013; Li et al., 2017; Yuan et al., 2018; Wang et al., 2020). Although some related genes or enzymes have been reported, the specific biosynthesis pathways of polysaccharides and alkaloids and even dendrobine biosynthesis in *Dendrobium* species remain unclear.

With omics technology development, many medicinal plant genomes have been sequenced, such as *Dendrobium catenatum* (Zhang et al., 2016), *Salvia miltiorrhiza* (Xu et al., 2016), *Panax Notoginseng* (Chen et al., 2017), *Gelsemium elegans* (Liu et al., 2020), and *Platycodon grandifloras* (Kim et al., 2020), suggesting that genomics is an effective method to mine the key genes of medicinal ingredients. In this study, with the use of PacBio sequencing and Hi-C technologies, the chromosome-level genome assembly of *D. nobile* was performed. Comparative genomic studies were conducted with *D. catenatum* (Zhang et al., 2016), *Dendrobium chrysotoxum* (Zhang et al., 2021), *Apostasia shenzhenica* (Zhang et al., 2017), and *Phalaenopsis equestris* (Cai et al., 2015). The genes involved in the biosynthesis pathway of polysaccharides and alkaloids (dendrobine) were identified in the present study, laying a foundation for further research on the gene functions of medicinal active ingredients and providing a reference for the breeding of new varieties. The metabolic pathway-related analysis in *Dendrobium* will provide further insight into how *Dendrobium* species respond to the environment.

## Materials and Methods

### Sample Preparation and Sequencing

The wild *Dendrobium nobile* (voucher specimen: China, Yunnan province, on rock in evergreen broad-leaf forest, alt. 1250 m, 15 April, 2019, GZMU001) plants were collected. The species was identified by comparison with the *D. nobile* specimen deposited in the herbarium of the National Orchid Conservation Center of China. Green young leaves were frozen for short reads sequencing and PacBio sequencing. Leaf buds from the plants were fixed as described in Wang et al., 2015a and Hi-C library construction is referred to Chen et al. (2020).

For short reads sequencing, genomic DNA was extracted from leaves of the *D. nobile* plants using a modified cetyltrimethylammonium bromide (CTAB) protocol, and then was shorn by Covaris ultrasonicator. DNA fragments between 300bp and 400bp were then selected by Agencourt AMPure XP-Medium kit for library construction and finally sequencing was performed on the MGISEQ-2000 platform. The raw data was generated from constructed paired-end libraries (PE150), and the clean data was obtained after data filtering, which was carried out by SOAPnuke v1.6.5 software (https://github.com/BGI-flexlab/SOAPnuke) (Chen et al., 2018), with the following parameters: -n 0.02; -l 20; -q 0.4; -Q 2; -i; -G; --seqType 0; –rmdup. The PacBio sequencing was performed on a PacBio Sequel II sequencer by BGI (Shenzhen, China). Furthermore, the data of Hi-C library sequenced by MGISEQ-2000 platform was used for Hi-C analysis.

For assisting gene annotation and gene expression analysis, total RNA was extracted from tender leaves, stems, and roots of three different individuals in the same growing stage of *D. nobile* using the RNAprep Pure Plant Kit and genomic DNA contamination was removed using RNase-Free DNase I (both from Tiangen), respectively. The integrity of RNA was evaluated on a 1.0% agarose gel stained with ethidium bromide (EB), and its quality and quantity were assessed using a Qubit2.0 Fluorometer and an Agilent 2,100 Bioanalyzer (Agilent Technologies). As the RNA integrity number (RIN) was greater than 7.0 for all samples, they were used in cDNA library construction and Illumina sequencing. The cDNA library was constructed using the NEBNext Ultra RNA Library Prep Kit for Illumina (NEB) and 3 μ g RNA per sample, following the manufacturer’s recommendations. The PCR products obtained were purified (AMPure XP system) and library quality was assessed on the Agilent Bioanalyzer 2,100 system. Library preparations were sequenced on the Illumina Novaseq6000 sequencer, generating 150-bp paired-end reads.

### Genome Assembly

Before genome assembly, genome size and heterozygosity were estimated by Jellyfish v.2.1.4 (Marcais and Kingsford, 2011) and GenomeScope (Vurture et al., 2017) based on a 17-K-mer distribution. Then, Canu v 2.2 (Koren et al., 2017) was used to correct the Pacbio raw data and assemble the genome with the following parameters: correctedErrorRate = 0.035 utgOvlErrorRate = 0.065 trimReadsCoverage = 2 trimReadsOverlap = 500 gridOptions = "--mem-per-cpu = 5 g”. Furthermore, pilon v1.22 (--fix bases--mindepth 10 --minqual 20 --diploid) (Walker et al., 2014) was used to correct the assembly using the data generated from the MGISEQ-2000 platform. For chromosome-level assembly, the Hi-C reads were filtered by SOAPnuke software (Chen et al., 2018) with the following parameters: -n 0.02; -l 20; -q 0.4; -Q 2; -i; -G; --seqType 1; –rmdup. Then, the obtained clean reads were compared with the preassembled contigs using Juicer software (Durand et al., 2016). After filtering the results and removing the misaligned reads, 3D-DNA (Dudchenko et al., 2017) software was used to preliminarily cluster, sequence, and direct the pseudochromosomes. Furthermore, Juicer-box was used to adjust, reset, and cluster the pseudochromosomes, and misassemblies and misjoins were manually corrected based on neighboring interactions. For the evaluation of Hi-C assembly results, the final pseudochromosome assemblies were divided into 150 kb bins with equal lengths, and the interaction signals generated by the valid mapped read pairs between each bin were visualized in a heat map. Finally, the completeness and quality of the final assembled genome were evaluated through Benchmarking Universal Single-Copy Ortholog (BUSCO v5.2.2) (Manni et al., 2021) tests.

### Genome Annotation

Repetitive sequences are an important part of the genome and are divided into two types: tandem and interspersed repeats. RepeatMasker v4.0.7 and RepeatProteinMask v4.0.7 software (http://www.repeatmasker.org) were used to identify repetitive sequences based on the RepBase v21.12 database (http://www.girinst.org/repbase). For *de novo* prediction, a repetitive sequence database was constructed through RepeatModeler (http://www.repeatmasker.org/RepeatModeler/) and LTR_FINDER v1.06 (http://tlife.fudan.edu.cn/ltr_finder/). RepeatMasker software was then used to predict the repeat sequences. Tandem Repeats Finder v4.09 (http://tandem.bu.edu/trf/trf.html) was used to find tandem repeats in the genome. The repeat-masked genome assembly was used for annotating high-quality protein-coding genes through an integration of homology-based, *de novo*, and transcriptome-based predictions. For homology-based prediction, protein sequences from seven species (*Arabidopsis thaliana*, *Oryza sativa*, *Asparagus officinalis*, *A. shenzhenica*, *Gastrodia elata*, *P. equestris, and Vanilla planifolia*) were used to align the *D. nobile* genome sequences through Genewise v2.4.1 (Birney et al., 2004) (https://www.ebi.ac.uk/Tools/psa/genewise/). Then, 5,000 complete genes from the homology-based prediction method were used to produce a training model using the Augustus (Stanke et al., 2006) (http://bioinf.uni-greifswald.de/augustus/) and SNAP (Johnson et al., 2008) (http://homepage.mac.com/iankorf/) software. The RNA sequencing data of *D. nobile* were mapped to the genome sequences through Hisat v2.1.0 and StringTie v1.3.4d (Kim et al., 2015; Pertea et al., 2015). Finally, Maker v2.31.8 (Holt and Yandell, 2011) was used to annotate and integrate the results produced by the three methods. BUSCO v5.2.2 (Manni et al., 2021) was used to evaluate the completeness and quality of the gene models.

Functional annotation of the predicted gene models was carried out by Blast v2.2.26 (Altschul, 1990) software, aligned against the Swissprot (Boeckmann et al., 2003) (http://www.uniprot.org/), TrEMBL (http://www.uniprot.org/), Kyoto Encyclopedia of Genes and Genomes (KEGG) (http://www.genome.jp/kegg/), InterPro (Zdobnov and Apweiler, 2001) (https://www.ebi.ac.uk/interpro/), Nr (non-redundant database), KOG (Koonin et al., 2004) (clusters of euKaryotic Orthologous Groups), and Gene Ontology (GO) (Ashburner et al., 2000) databases. For non-coding RNA annotation, tRNAscan-SE 1.3.1 (Lowe and Eddy, 1997) (http://lowelab.ucsc.edu/tRNAscan-SE/) was used to annotate the tRNA sequences. BLASTN was used to search for rRNA. The miRNA and snRNA sequences were predicted by the INFERNAL (Griffiths-Jones, 2004) (http://infernal.janelia.org/) software.

### Gene Family Identification

The protein sequences of *D. nobile* and 17 additional angiosperm species (*D. catenatum, D. chrysotoxum, Cymbidium sinense, Amborella trichopoda, Spirodela polyrhiza, A. shenzhenica, A. officinalis, O. sativa, Ananas comosus, Musa acuminata, Phoenix dactylifera, Vitis vinifera, P. equestris, Sorghum bicolor, Populus trichocarpa, A. thaliana,* and *G. elata*) were used for orthologous gene family identification and clustering. BLASTP was used to calculate the similarities between sequence pairs with a cut-off e value of 1e^−5^. Finally, OrthoMCL v.2.0.9 (Li et al., 2003) with default parameters was used to cluster the gene families.

### Phylogenetic Tree Construction and Phylogenomic Dating

The protein sequences of each gene family of 313 identified single-copy gene families were aligned by MUSCLE (Edgar, 2004) (http://www.drive5.com/muscle/). Then, a super-alignment matrix generated from all the alignment results was used for the construction of the phylogenetic tree. Finally, RAxML (Stamatakis, 2014) was used for the construction of the phylogenetic tree of 18 angiosperm species with the GTRGAMMA model; the bootstrap value was 1,000.

PAML MCMCTREE (http://abacus.gene.ucl.ac.uk/software/paml.html) was used to estimate the divergence time. The following constraints were used for time calibrations: i) the *B. distachyon* and *O. sativa* divergence time [40.0–54.0 million years (Ma)] (International Brachypodium Initiative, 2010); ii) the divergence time of *P. trichocarpa* and *A. thaliana* (100.0–120.0 Ma) (Tuskan et al., 2006); iii) the monocot and eudicot divergence time (lower boundary of 130.0 Ma) (Jaillon et al., 2007); and iv) the time of the earliest-diverging angiosperms (<200.0 Ma) (Magallón et al., 2013).

### Whole-Genome Duplication and Collinearity Analysis

The *K*s distribution was used to infer whole-genome duplication (WGD) events in *D. nobile* based on paralogous gene pairs and the divergence between species based on orthologues. MCscanX v1.5.2 (Wang et al., 2012) was used to find the collinear regions. BLASTP was used to search for putative paralogous genes in each collinear region within *D. nobile* and orthologous genes between *D. nobile* and *D. chrysotoxum*, *D. nobile* and *V. planifolia*, and *D. nobile* and *C. sinense*. Furthermore, Codeml, in the PAML package (Yang, 1997) was used with the F3X4 model to calculate the *Ks* value of each gene pair.

For further analysis of the features of collinear regions, MCscan (https://zenodo.org/record/31631#.XpkUyTOeask) was used to find the collinear regions between *D. nobile* and *D. chrysotoxum*, *D. nobile* and *V. planifolia*, and *D. nobile* and *C. sinense*.

### Expansion and Contraction of Gene Families

Based on the gene families clustered by OrthoMCL (Li et al., 2003), we filtered the gene families with a number higher than 200 in one species and lower than 2 in other species. CAFÉ software (De Bie et al., 2006) (http://sourceforge.net/projects/cafehahnlab/) was used to determine the expansion and contraction of orthologous gene families combined with divergence times.

### Gene Family Analysis of the Biosynthesis of Bioactive Components

All the genes or gene families in the biosynthesis pathways of polysaccharides and alkaloids were identified by HMM or BLASTP searches. The HMM profiles (PF01128.20 for *CMS* genes, PF01264.22 for *CS* genes, PF13292.7 for *DXS* genes, PF02401.19 for *HDR* genes, PF04551.15 for *HDS* genes, PF08540.11 and PF01154.18 for *HMGS* genes, PF00288.27 for *PMK* genes, PF00275.21 for *SHKG* genes, PF01202.23 for *SK* genes, and PF03088.17 for *STR* genes) were downloaded from Pfam (pfam.xfam.org/), and the query sequences for the BLASTP methods were mostly from homologous genes of *Arabidopsis*, except for the *SKDH* genes from *Pisum sativum* (Weeden, 2018) and *DHQS* genes from tomatoes (Bischoff et al., 1996). After the candidate homologs were obtained, MAFFT (Katoh and Standley, 2013) and PhyML (Guindon et al., 2005) software were used for sequence alignment and phylogenetic tree construction, respectively.

### *TPS* and *CYP450* Gene Family Identification

The HMM profiles for PF01397 (Terpene_synth) and PF03936 (Terpene_synth_C) were downloaded from Pfam (pfam.xfam.org/), and two profiles were used to carry out HMM searches against the protein database for six species (*D. nobile*, *D. chrysotoxum*, *D. catenatum*, *P. equestris*, *A. shenzhenica*, and *A. thaliana*). These sequences were then manually checked, and the sequences with at least one of these domains were retained. The retained amino acid sequences were aligned using MAFFT (Katoh and Standley, 2013). Then, the aligned amino acids were used for phylogenetic tree construction by PhyML (Guindon et al., 2005). The tree was generated by the maximum likelihood method based on the Jones-Taylor-Thornton (JTT) matrix-based model (Jones et al., 1992) and the bootstrap method for phylogeny tests with 1,000 replications. The HMM profiles for PF00067.23 (CYP450) were downloaded from Pfam (pfam.xfam.org/). HMM searches were used to obtain the homologs in the protein database for seven species (*D. nobile*, *D. chrysotoxum*, *D. catenatum*, *P. equestris*, *A. shenzhenica*, *A. thaliana*, and *O. sativa*). The next steps were the same as those for *TPS* gene family identification, except for the rice genome. The identified *CYP450* genes in *A. thaliana* and *O. sativa* in this study were further confirmed with the *Arabidopsis* Cytochrome P450 database (http://www.p450.kvl.dk/At_cyps/family.shtml) and rice (Wei and Chen, 2018). All gene expression analyses were carried out using Salmon v1.3.0 (Patro, et al., 2017) with the default settings.

## Results

### Genome Sequencing and Genomic Characteristics

**FIGURE 1:**
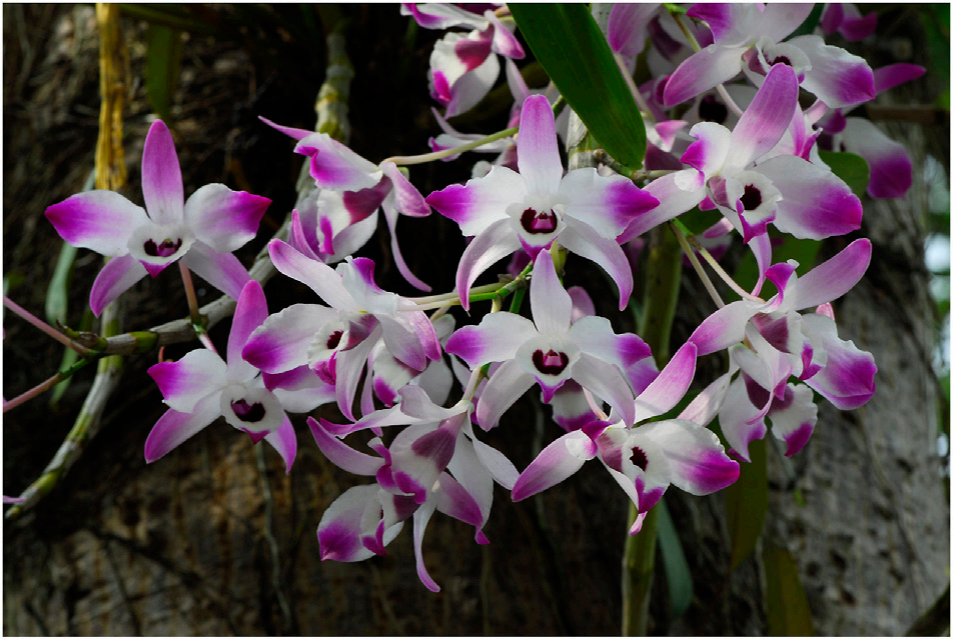
The plant of *Dendrobium nobile*.

**FIGURE 2:**
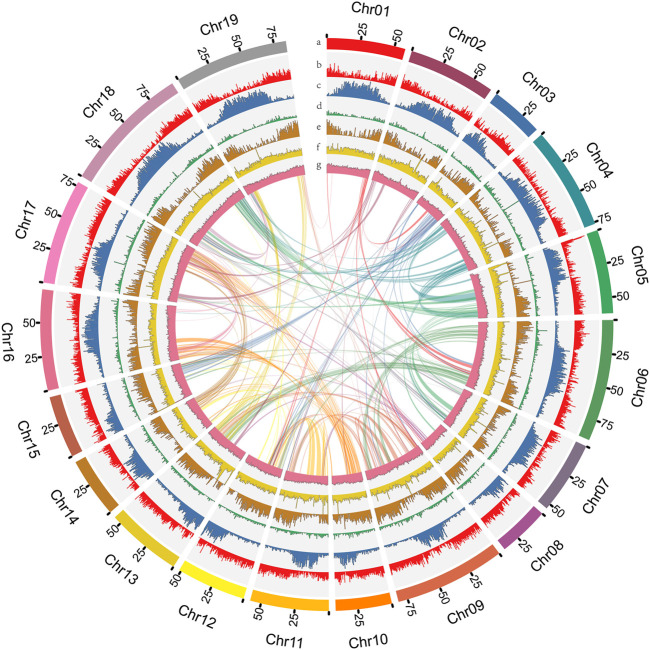
The genome features of *Dendrobium nobile*. **(A)** Chromosomes. **(B)** LTR Gypsy density. **(C)** LTR Copia density. **(D)** DNA transposon density. **(E)** Gene density. **(F)** Coverage of second-generation data. **(G)** GC content within sliding windows of 500 kb.

**FIGURE 3:**
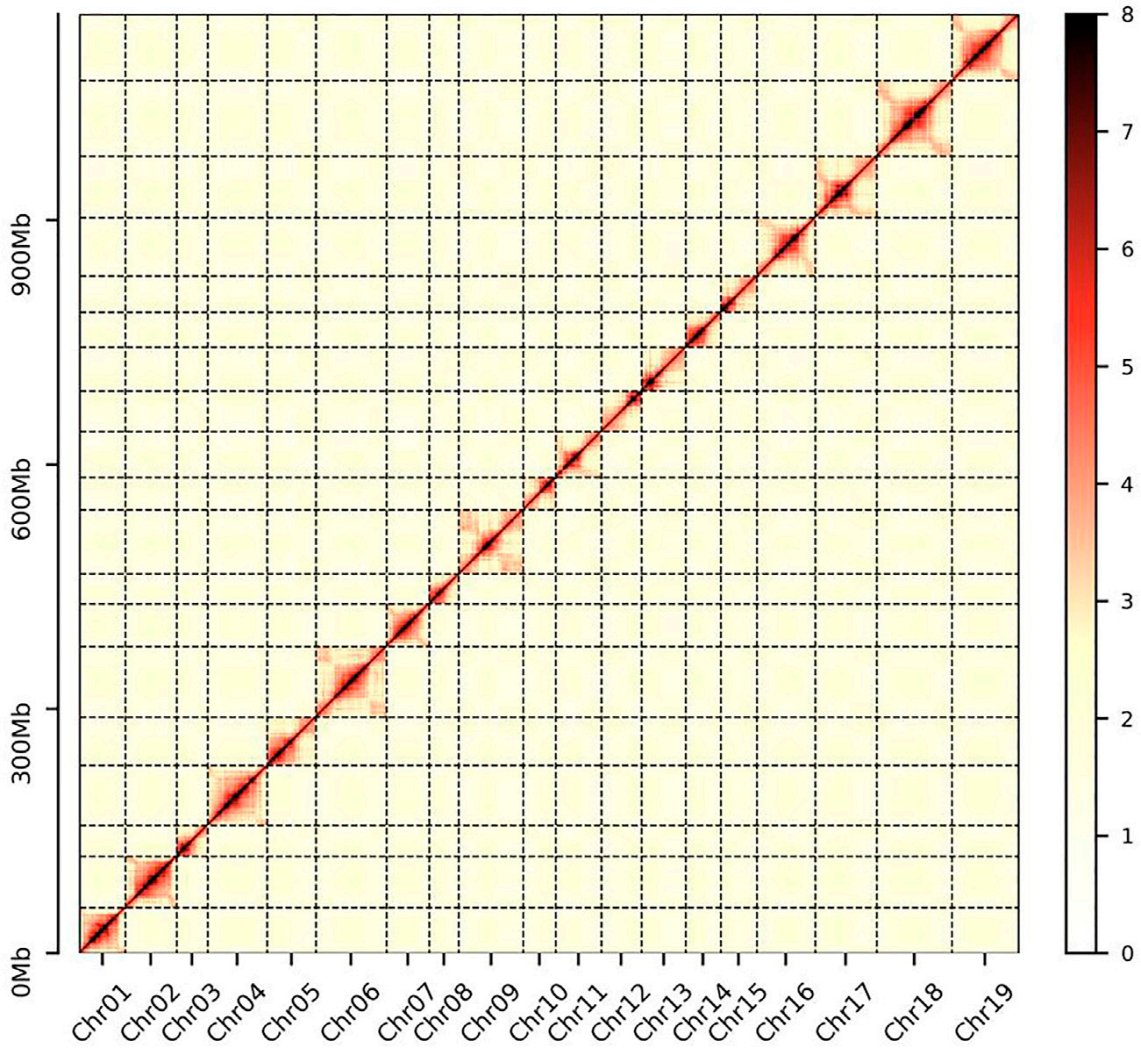
Intensity signal heat map of the Hi-C chromosome. A higher value on the scale bar indicates a higher contact frequency.

*D. nobile* (Figure 1) has a karyotype of 2*N* = 2X = 38 (Zheng et al., 2018). For estimating the *D. nobile* genome size, a total of 130.62 Gb of raw data with 300-400 bp insert libraries were generated by MGISEQ-2000 sequencing and 122.52 Gb of clean data was obtained after data filtering (Supplementary Table S1). The estimated genome size was 1.16 Gb, with 1.35% heterozygosity based on *K-mer* analysis (Supplementary Figure S1). To obtain the assembly, 96.91 Gb (coverage of 83.54×) of PacBio sequencing data and 5.19 million subreads with an N50 read length of 21.4 kb was generated (Supplementary Table S1). Based on the PacBio and MGISEQ-2000 sequencing data, we then used 245.48 Gb of raw data from a Hi-C library (Supplementary Table S1) to reconstruct physical maps by recoding and clustering the assembled scaffolds into 19 pseudochromosomes, which represented the 19 chromosomes in the haploid genome of *D. nobile* (Figure 2; Supplementary Table S2). Finally, 211.34 Gb of clean data was produced using the data filtering. After the Hi-C analysis, 1,192,377,146 bp sequences were mapped to 19 pseudochromosomes, accounting for 99.45% of the raw assembly (1.19 Gb). The length of the 19 pseudochromosomes ranged from 37.78 to 95.36 Mb, with a scaffold N50 value of 64.46 Mb (Table 1; Supplementary Tables S2, S3). Furthermore, BUSCO estimation indicated that the completeness of the gene set of the assembled genome was 96.22%, while mapping short reads back to the assembly indicated that the completeness was 98.82% (Supplementary Tables S4, S5). The chromatin interaction data showed a high level of chromatin interaction between linked sequences and low chromatin interaction between non-linked sequences, suggesting the high quality of the Hi-C assembly (Figure 3).

**TABLE 1:** Genome assembly statistics.

Assembly size (bp)	1,199,116,975
GC Content (%)	35
Number of scaffolds	57
Longest scaffold (bp)	95,358,005
Contig N50 (bp)	1,618,306
Scaffold N50 (bp)	64,459,299
Sequence in chromosomes (%)^a^	99.45
Complete BUSCOs (%)	96.22%
Mapping statistics of short reads	98.82

a Sequences in the 19 described pseudochromosomes.

### Gene Prediction and Annotation

A total of 29,476 protein-coding genes were annotated in *D. nobile* (Supplementary Table S6). Among them, 98.28% of ≥50% CD regions overlapped based on homolog or *de novo* data (Supplementary Table S7), with 93.43% exhibiting high completeness (Supplementary Table S8).

The average lengths of genes and introns in *D. nobile* were similar to those in other orchid species, except for *A. shenzhenica* (Zhang et al., 2017), and much higher than those in most other angiosperms (Zhang et al., 2017; Supplementary Figure S2). This suggests that the gene length is positively correlated with the intron length, and that genes and introns in orchids except for *A. shenzhenica* have a longer average length, which may be a unique characteristic. Furthermore, 76 microRNAs, 386 transfer RNAs, 958 ribosomal RNAs, and 457 small nuclear RNAs were identified in the *D. nobile* genome (Supplementary Table S9).

In this study, it was estimated that 61.07% of the *D. nobile* genome consisted of repetitive sequences (Supplementary Table S10), similar to the 62% in *P. equestris* and 62.81% in *D. chrysotoxum*, and lower than the 78.1% in *D. catenatum* (Cai et al., 2015; Zhang et al., 2016). Furthermore, the notably higher percentage of repeats predicted by the *de novo* method compared to the homology-based search method indicates that *D. nobile* has many characteristic repeats (Supplementary Table S10). Among these elements, long terminal repeats (LTRs) accounted for 51.31% of the genome (Supplementary Table S11), similar to the 53.15% in *D. chrysotoxum* and higher than the 46% in *D. catenatum*, 46.47% in *P. equestris*, and 22.06% in *A. shenzhenica* (Zhang et al., 2016; Zhang et al., 2017).

In addition, 27,765 (94.20%) predicted genes were functionally annotated (Supplementary Table S12). Among them, 27.601 (93.64%) and 25,870 (87.77%) genes were annotated to the Nr and TrEMBL databases, respectively, (Supplementary Table S14). The number of annotated genes was 24,044 (81.57%), 20,215 (68.58%), and 19,855 (67.36%) in the Interpro, KEGG, and Swissprot databases, respectively (Supplementary Table S12).

### Evolution of Gene Families

**FIGURE 4:**
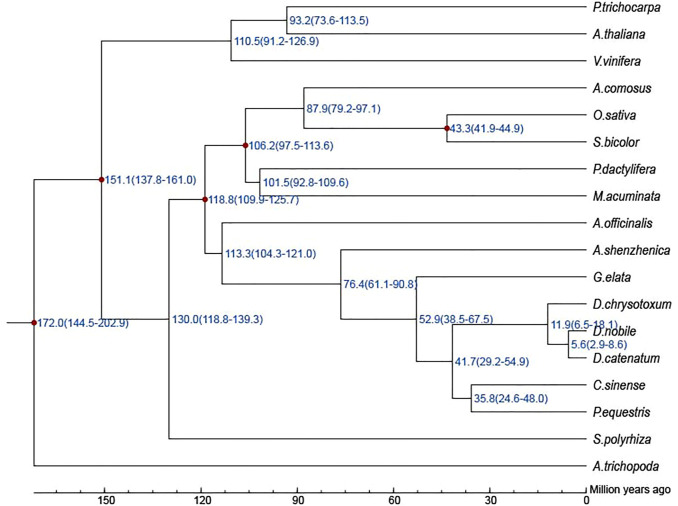
Divergence time among 19 species.

**FIGURE 5:**
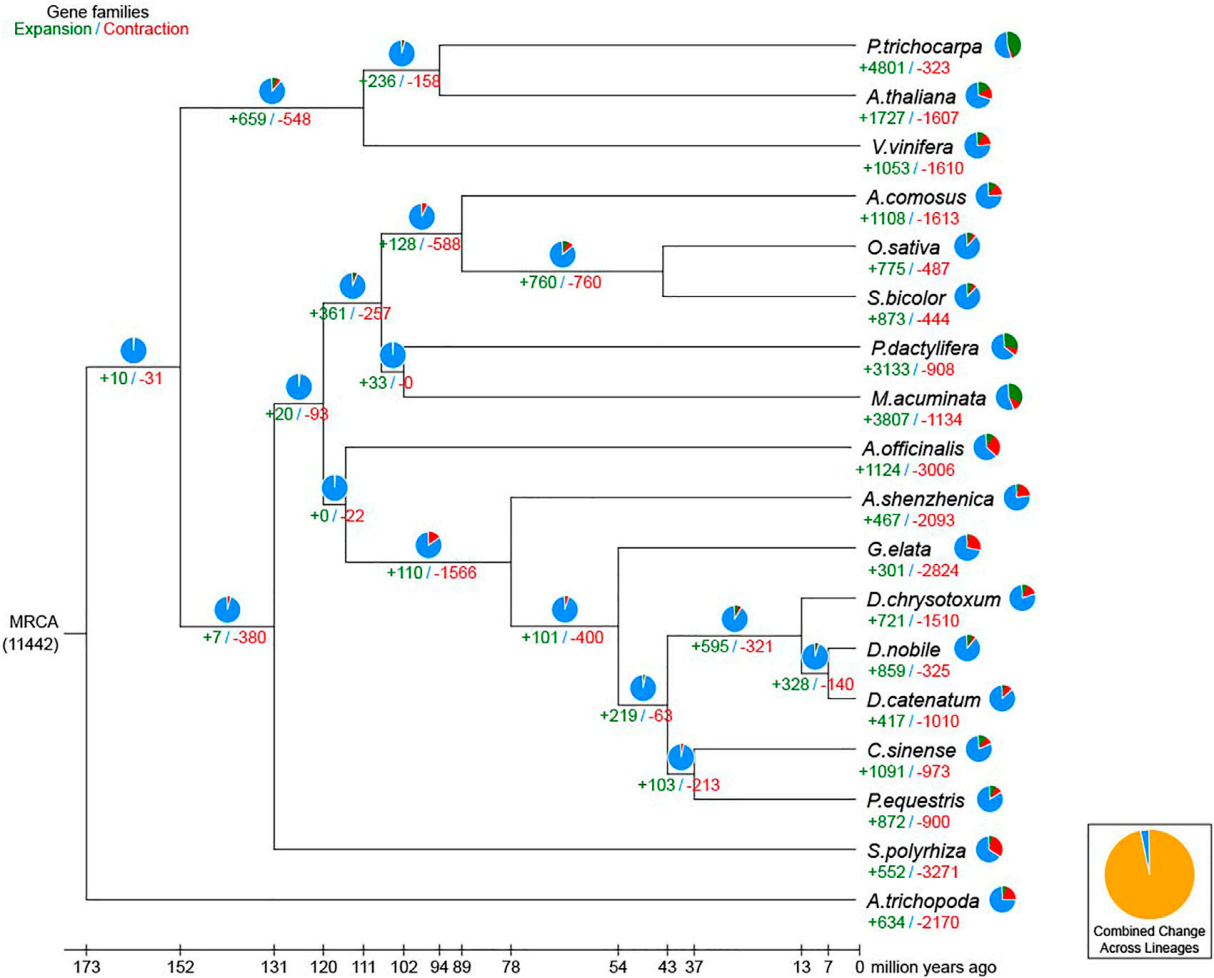
Expansion and contraction of gene families. The green and red numbers are the numbers of expanded and contracted gene families, respectively.

For the phylogenetic relationship and divergence times among different plant species, a high-confidence phylogenetic tree and the estimated divergence times of 18 different plant species based on genes extracted from a total of 313 single-copy families were constructed (Supplementary Figures S3, S4; Supplementary Table S13). As expected, *D. nobile*, *D. catenatum*, and *D. chrysotoxum* were sisters to *P. equestris*, forming an Epidenroideae clade. *G. elata* and *A. shenzhenica* were located at the base of the Orchidaceae branches (Supplementary Figure S4). The estimated Orchidaceae divergence time was 113.3 (104.3–121.0) Mya, the divergence time of the subfamily Apostasioideae was 76.4 (61.1–90.8) Mya, and the divergence time between *D. nobile* and *P. equestris* was 41.7 (29.2–54.9) Mya (Figure 4). Finally, *D. chrysotoxum* appeared at 11.9 (6.5–18.1) Mya, and the divergence time between *D. nobile* and *D. catenatum* was 5.6 (2.9–8.6) Mya. Then, the expansion and contraction of orthologous gene families were determined. According to the results (Figure 5), 110 gene families were expanded in the lineage leading to the Orchidaceae, whereas 1,566 gene families were contracted. Furthermore, 595 gene families expanded and 321 gene families contracted, leading to the *Dendrobium* genus. In *D. nobile,* 859 gene families were expanded compared with 417 in *D. catenatum*, 721 in *D. chrysotoxum*, and 872 in *P. equestris*. For the contracted gene families, 325 gene families were contracted in *D. nobile* compared with 1,010 in *D. catenatum*, 1,510 in *D. chrysotoxum*, and 900 in *P. equestris*. In the *D. nobile* clade, 859 gene families were expanded, including 3,134 genes, and 325 gene families were contracted, including 213 genes. To further investigate the functions of the expanded gene families and their species specificity, KEGG enrichment analysis was conducted for the expanded gene families. The KEGG term “Biosynthesis of other secondary metabolites” was found to be the most significantly enriched, and contained the most genes (Supplementary Table S14). This may be related to the synthesis of specific medicinal ingredients in *D. nobile*.

### Whole-Genome Duplication and Synteny Analysis

**FIGURE 6:**
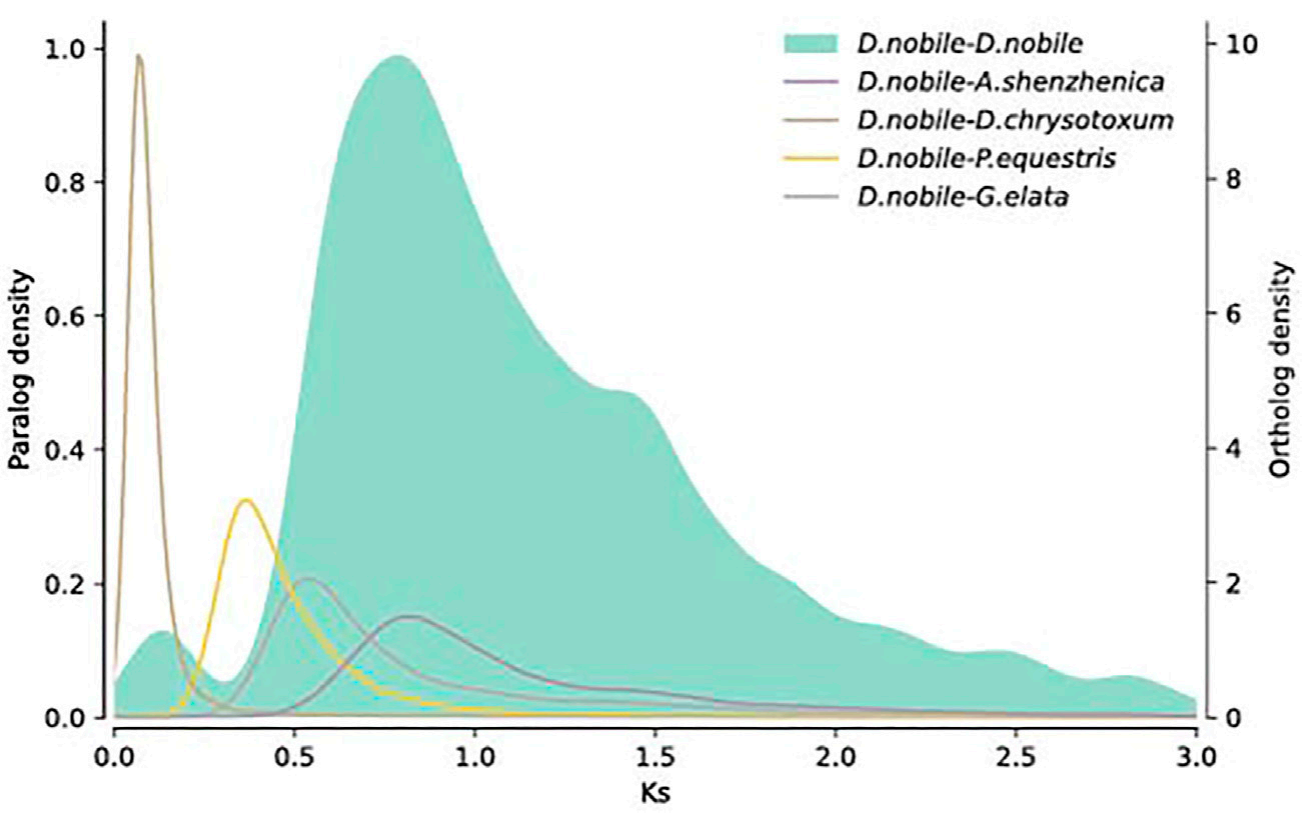
Distribution of *Ks* of the whole paranome of *Dendrobium nobile*. The *Ks* distribution of paralogues in the *D. nobile* genome is shown in the gray histogram and gray density curve. The other density curves show the *Ks* distribution of orthologous genes between *D. nobile* and *Phalaenopsis equestris*, *D. nobile* and *Apostasia shenzhenica*, *D. nobile* and *Dendrobium chrysotoxum*, and *D. nobile*, and *Gastrodia elata*.

To detect the occurrence of WGDs in *D. nobile*, the *Ks* distribution pattern of paralogous genes in collinear regions was analyzed. There were two peaks, and the *Ks* values were 0.8 and 1.5 (Figure 6), suggesting the occurrence of two recent WGD events in *D. nobile*. To determine the time of the two WGD events, the *Ks* distribution of the homologous genes between *D. nobile* and *P. equestris*, *A. shenzhenica*, *D. chrysotoxum*, and *G. elata* was further analyzed*.* The most recent WGD event in *D. nobile* (*Ks* = 0.8) occurred before the divergence between *D. nobile* and *A. shenzhenica*, suggesting that the WGD events were shared among all extant orchids (Zhang et al*.*, 2017). The second recent WGD event in *D. nobile* (*Ks* = 1.5) was shared with most monocots (Zhang et al., 2017). For the collinear regions, the syntenic relationships among *D. nobile*, *V. planifolia*, *C. sinense*, and *D. chrysotoxum* were further analyzed. There were 25,166 collinear gene pairs located in 1,404 collinear regions between *D. nobile* and *V. planifolia.* 31,498 collinear gene pairs located in 120 collinear regions between *D. nobile* and *D. chrysotoxum*, and 30,650 collinear gene pairs located in 430 collinear regions between *D. nobile* and *C. sinense* (Supplementary Figures S5–S7; Supplementary Table S15).

### Genes in the Polysaccharide Biosynthesis Pathway

**FIGURE 7:**
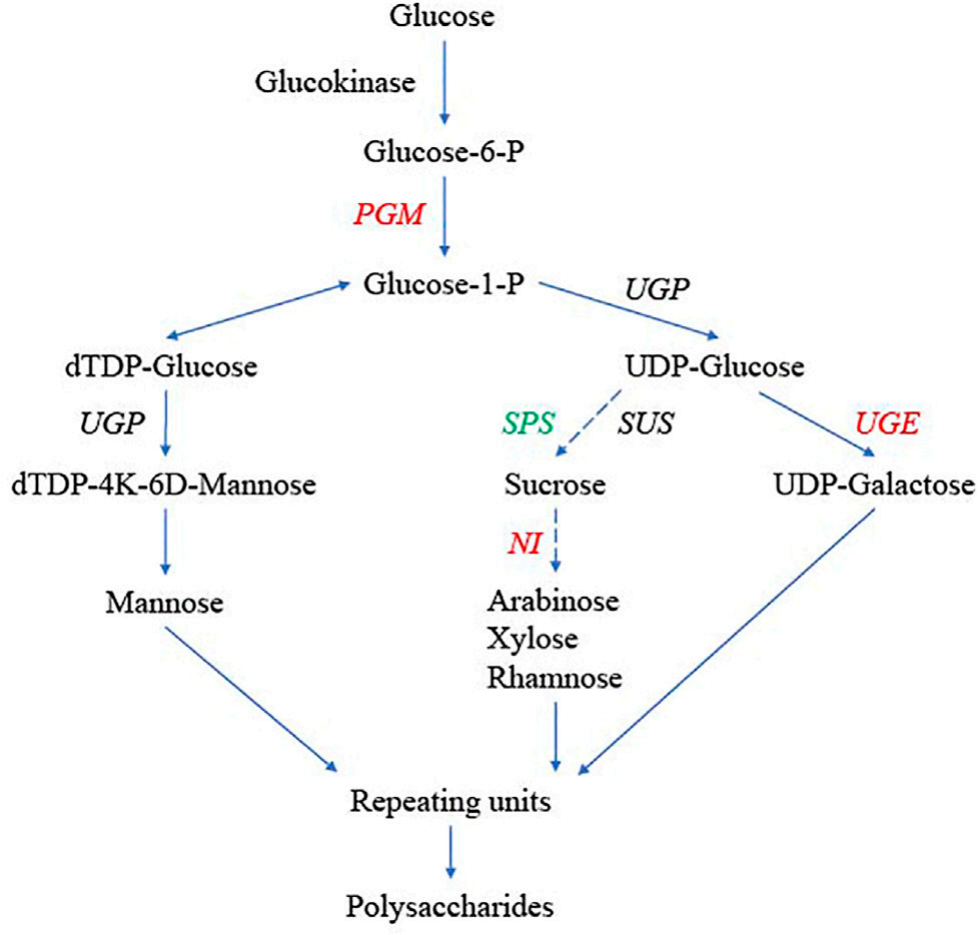
Genes in the polysaccharide synthesis pathway in *Dendrobium nobile*, adapted from Wang (Wang et al., 2020). Hydrolysis or hydrolysis-derived reactions could generate monosaccharides, such as mannose, glucose, and galactose. These monosaccharides are also the basic building blocks and repeating units for synthesizing polysaccharides. Genes in red indicate gene copy number variation among *D. nobile*, *Dendrobium catenatum*, *Dendrobium chrysotoxum*, *Phalaenopsis equestris*, *Apostasia shenzhenica*, and *Arabidopsis thaliana*. Genes in green are *Dendrobium-*specific. The dashed lines indicate multiple steps.

The key genes encoding enzymes in the potential polysaccharide synthesis pathway were identified in *D. nobile*, *D. catenatum*, *D. chrysotoxum*, *A. shenzhenica*, *P. equestris*, and *A. thaliana* (Figure 7; Supplementary Table S16). All six gene families were multigene families. There were seven alkaline/neutral invertase (*NI*) genes, two *PGM* genes, three *SPS* genes, four *SUS* genes, two *UGP* genes, and five *UGE* genes in *D. nobile*. Based on the phylogenetic analysis, these gene families could be further divided into several branches. The number of genes in different branches among these species varied greatly, especially those in the *NI*, *PGM*, and *UGE* gene families (Figure 7, in red font). The number of *NI* genes in the cytosolic branch, mitochondria branch, and chloroplastic branch of *D. nobile* was five, two, and one, respectively, compared with seven, one, and one in *D. chrysotoxum*, respectively, and three, three, and one in *D. catenatum*, respectively (Supplementary Table S17). For *PGM* genes, one gene in *D. nobile* was clustered into the pPGM branch, and one gene were divided into the cPGM branch (Supplementary Table S16). Interestingly, there were five *PGM* genes of *D. chrysotoxum* on the cPGM branch and one gene on the pPGM branch (Supplementary Table S16). There were five and zero members divided into group 1 and group 2 in the *UGE* gene family, respectively, while there were one to two different values among other species (Supplementary Table S16). The gene number variations in different branches of each gene family may be related to the different amounts and constituent polysaccharides among *Dendrobium* species. Furthermore, for the SPS4F branch in the *SPS* gene family, three homologous genes were identified in three *Dendrobium* species, and no genes were found in other orchid species (*A. shenzhenica* and *P. equestris*), suggesting a *Dendrobium*-specific gene (Figure 7, in green font; Supplementary Table S16). The gene expression patterns in different tissues of the three *Dendrobium* species were also analyzed, but most were not significantly different among tissues.

### Genes Upstream of the Alkaloid Biosynthesis Pathway

**FIGURE 8:**
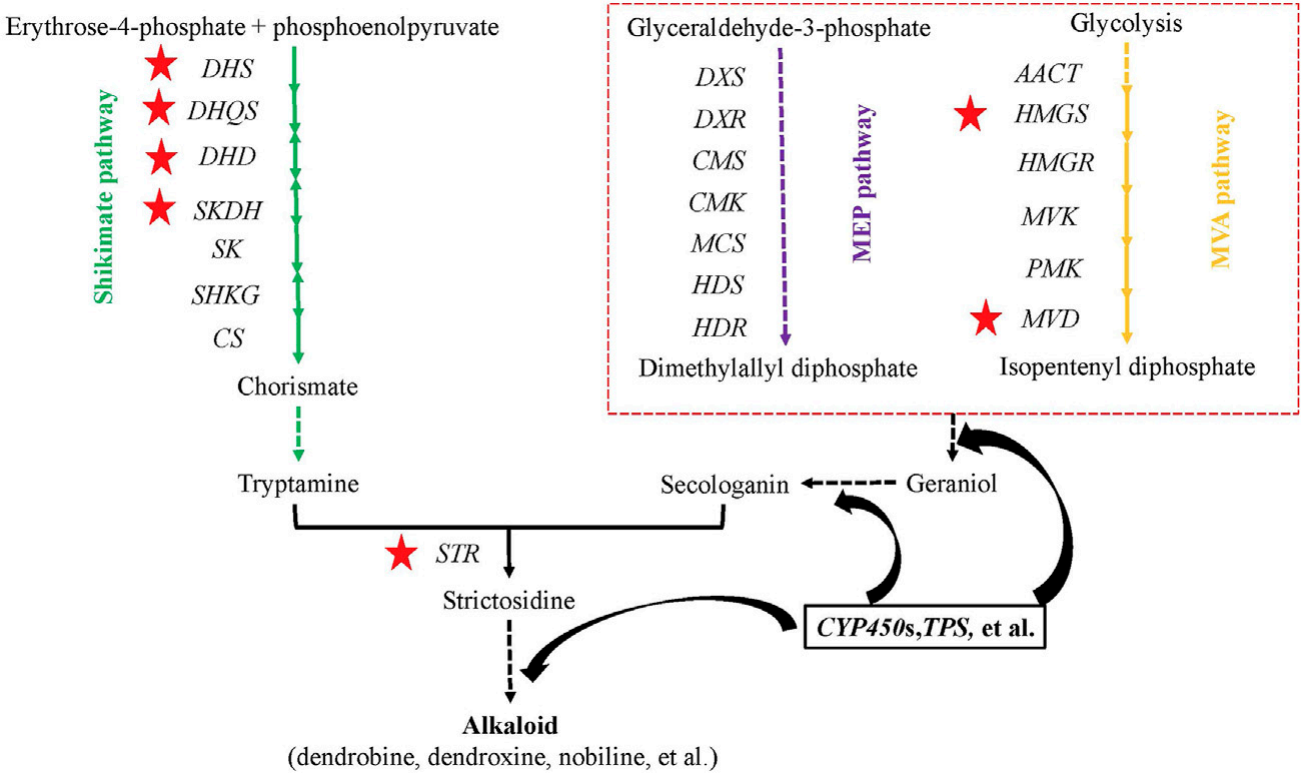
Genes upstream of the alkaloid biosynthesis pathway in *Dendrobium nobile,* adapted from Pu (Pu et al., 2021) and Wang (Wang et al., 2020). In the methylerythritol phosphate (MEP) pathway, there are seven consecutive steps catalyzed by the following enzymes: DXS, 1-deoxy-D-xylulose 5-phosphate (DXP) synthase; DXR, DXP-reductoisomerase; MCT, 2-C-methyl-D-erythritol 4-phosphate cytidylyltransferase; CMK, 4-(cytidine 5′-diphospho)-2-C-methyl-D-erythritol kinase; MDS, 2-C-methyl-D-erythritol 2,4-cyclodiphosphate synthase; HDS, 4-hydroxy-3-methylbut-2-enyldiphosphate (HMBPP) synthase; and HDR, HMBPP reductase. The mevolonate (MVA) pathway contains ACCT, acetyl-coenzyme A (CoA) *C*-acetyltransferase; HMGS, HMG-CoA synthase; HMGR, HMG-CoA reductase; MVK, mevalonic acid kinase; PMK, phosphomevalonate kinase; and MVD, mevalonate-5-diphosphate decarboxylase. The shikimate pathway contains DHS, 3-dexoy-7-phosphoheptulonate synthase; DHQS, 3-dehydroquinate synthase; DHD, 3-dehydroquinate dehydratase; SKDH, shikimate dehydrogenase; SK, shikimate kinase; SHKG, 3-phosphoshikimate 1-carboxyvinyltransferase; and CS, chorismate synthase. The red five-pointed stars show higher gene expression in stems than in leaves. The dashed lines indicate multiple steps.

To determine the genetic variation of the alkaloid biosynthesis pathway, all key genes (20 genes or gene families) upstream of the alkaloid biosynthesis pathway were identified among three *Dendrobium* species (*D. nobile*, *D. catenatum*, and *D. chrysotoxum*) and three other species (*A. shenzhenica*, *P. equestris*, and *A. thaliana*) (Supplementary Table S17)). Most were single-copy genes, except for the *DHS*, *DXS*, and *HMGR* gene families. Interestingly, no *SKDH*, *HMGR*, or *MVD* genes were found in *D. chrysotoxum*, which hinted at a significant variation in genes or the possibility of alternative pathways playing a dominant role in this process. The gene expression patterns in different tissues among the three *Dendrobium* species were also determined. Three genes or gene families in the shikimate pathway exhibited higher expression in stems than in leaves: *Dnobile10G00647.1 (DHS)*, *Dnobile17G01430.1 (DHS)*, *Dnobile08G00887.1 (DHQS)*, and *Dnobile02G00904.1 (DHD-SKDH)* (Figure 8, red five-pointed star; Supplementary Figure S8). The *Dnobile08G00649.1 (HMGS)* and *Dnobile01G00682.1* (*MVD*) genes in the MVA pathway also had higher expression in stems (Figure 8, red five-pointed star). All genes in the MEP pathway were expressed equally in stems and leaves. For *D. chrysotoxum*, most genes had higher expression in leaves than in stems (Supplementary Figure S9), such as *Guchui-Maker62728* (*DHS*), *Guchui-Maker69398* (*DXR*), *Guchui-Maker111118* (*MCS*), *Guchui-Maker68853* (*HDS*), and *Guchui-Maker109159* (*HDR*). For *STR* genes, *Dnobile14G00298.1* (STR12/13) was mainly expressed in stems (Supplementary Figure S10), while other members mainly low or equally expression between the two tissues. Interestingly, *Guchui-Maker98640* (STR9) was mainly expressed in stems, and *Guchui-Maker96976* (STR10) was mainly expressed in leaves (Supplementary Figure S10). Dendrobine is mainly produced in the stems of *D. nobile* (Li et al., 2019) and is rare in *D. chrysotoxum*. These opposite gene expression patterns may be related to the difference between *D. nobile* and *D. chrysotoxum* in terms of alkaloid (dendrobine) biosynthesis.

### *TPS* Gene Family and Dendrobine Biosynthesis

Dendrobine, a sesquiterpenoid alkaloid, is the dominant type of alkaloid in *D. nobile*. TPSs catalyze the biosynthesis of sesquiterpenes (C15) using farnesyl diphosphate (FPP) as a substrate (McGarvey and Croteau, 1995). *TPSs* may have originated from isoprenyl diphosphate synthase genes, which are involved in dendrobine biosynthesis (Jiang et al., 2019; Wang et al., 2020). *CrGES* (a gene encoding geraniol synthase that is present in *Catharanthus roseus*, and is the homologue of *TPS02*) and *TPS21* genes are involved in dendrobine biosynthesis (Kuang et al., 2016; Wang et al., 2020).

**FIGURE 9:**
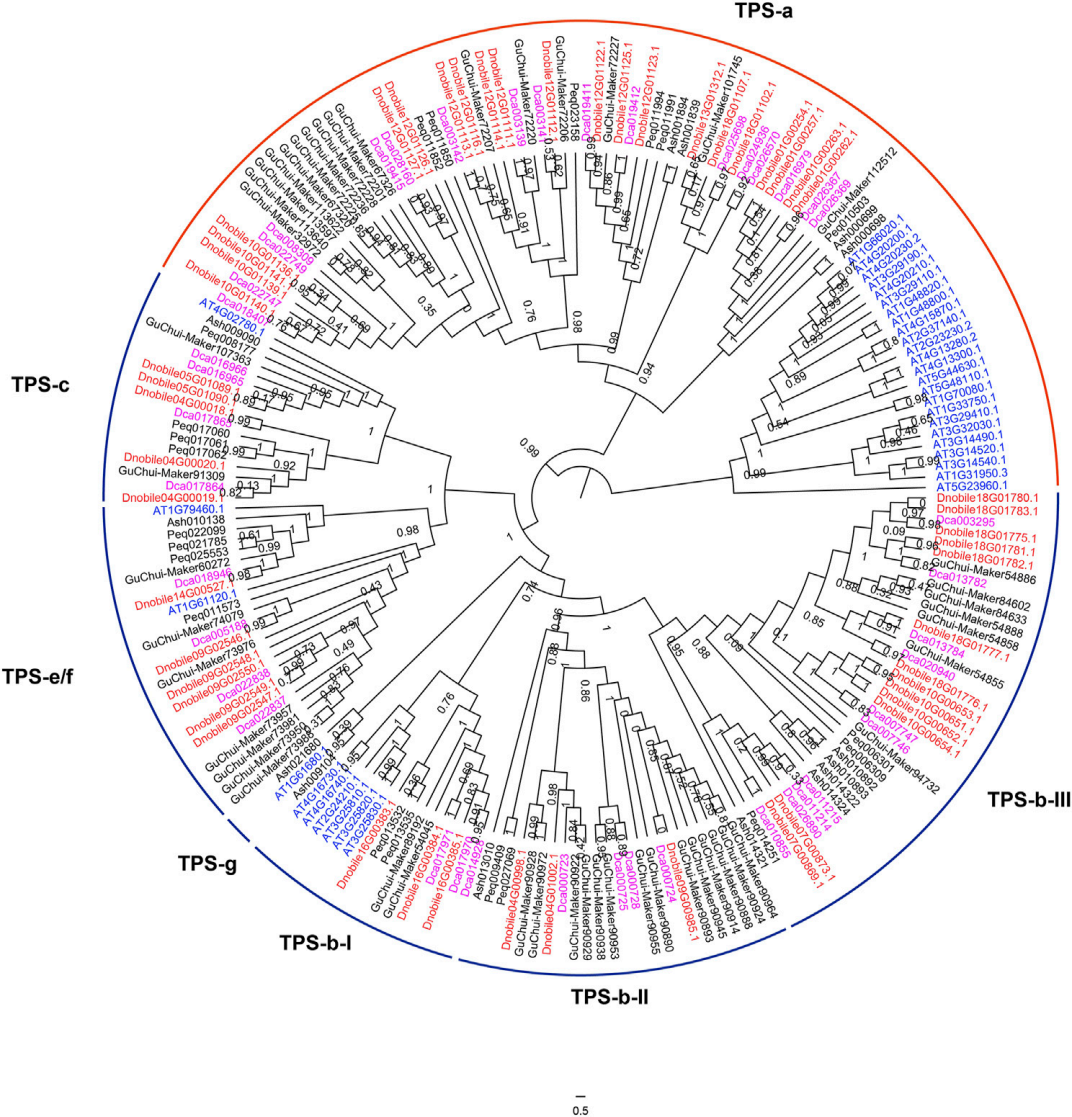
Analysis of terpene synthase (TPS) genes in *Dendrobium nobile*. Phylogenetic analysis of TPS genes in *D. nobile*, *Dendrobium chrysotoxum*, *Dendrobium catenatum*, *Apostasia shenzhenica*, *Arabidopsis thaliana*, and *Phalaenopsis equestris.* Dnobile, *D. nobile*; Ash, *A. shenzhenica*; Guchui-Maker, *D. chrysotoxum*; Dca, *D. catenatum*; Peq, *P. equestris*; AT, *A. thaliana*.

In this study, 51 *TPS* genes were identified in *D. nobile*, which was more than the 48 identified in *D. chrysotoxum* and the 42 identified in *D. catenatum* (Figure 9). The number of *TPS* genes in *Dendrobium* was much higher than that in *A. shenzhenica* and *P. equestris*, which indicated a relationship with dendrobine biosynthesis. The *TPS* gene family was divided into five subfamilies, TPS-a, TPS-b, TPS-c, TPS-e/f, and TPS-g, and the TPS-b subfamily was further clustered into TPS-b-I, TPS-b-II, and TPS-b-III. The TPS-a and TPS-b subfamilies showed different evolutionary patterns between monocots and dicots. Interestingly, there were 21, 16, and 17 TPS-a genes in *D. nobile*, *D. chrysotoxum*, and *D. catenatum*, respectively. As the TPS-a subfamily encodes only sesqui-TPSs (Jiang et al., 2019), a higher number of TPS-a genes in *D. nobile* may be an important factor in the higher production of dendrobine compared with other *Dendrobium* species (Li et al., 2019). Based on gene expression pattern analysis, genes of the TPS-a subfamily were mainly expressed in the stem (Supplementary Figure S11), which further confirmed that these genes contributed to the production of more dendrobine in *D. nobile* through the existence of more genes with higher expression in the stems. Furthermore, the gene numbers of TPS-b-II and TPS-b-III were three and 13 in *D. nobile*, respectively, compared with four and 10 in *D. catenatum*, respectively, while the numbers were 14 and seven in *D. chrysotoxum*, respectively. There were several *TPS* genes with higher expression in the leaves than in the stem (Supplementary Figure S11), such as *Dnobile18G01782.1*, *Dnobile18G01781.1*, *Dnobile18G01775.1*, and *Dnobile18G01780.1*. As the TPS-b subfamily encoding monoTPSs (Jiang et al., 2019), the opposite distribution and gene expression pattern may have contributed to the differences in the amounts and constitution of alkaloids between *D. nobile* and *D. chrysotoxum* in different tissues.

### *CYP450* Gene Family and Dendrobine Biosynthesis

Cytochrome P450 monooxygenases (CYP450s) are integral components in terpenoid and alkaloid pathways (Schuler, 2015). Biosynthetic CYP450s in these pathways can be considered organism-specific (Schuler, 2015). In this study, all the *CYP450* genes were identified in three *Dendrobium* species (*D. nobile*, *D. chrysotoxum*, and *D. catenatum*), two other orchid species (*P. equestris* and *A. shenzhenica*), *A. thaliana*, and rice. There were 123, 170, 228, 257, and 210 CYP450 gene members in *A. shenzhenica*, *P. equestris*, *D. nobile*, *D. chrysotoxum*, and *D. catenatum*, respectively. The number of genes in *Dendrobium* species was much higher than that in *P. equestris* and *A. shenzhenica*, mainly because of tandem gene duplication (Supplementary Figure S12).

CYP450 families are grouped into the 10 plant CYP450 clans (Nelson et al., 2004), and many duplications and divergence events, such as chemical defense pathways in particular plants or groups of plants, occur mainly in the expanded multiple-family clans. The species-specific synthesis and catabolism pathways of terpenoids are mainly caused by gene neofunctionalizations within the CYP85 clan (Schuler, 2015). In this study, the differences in CYP450 genes among species were also mainly detected in the CYP85 clan. The CYP720 subfamily, which mediates the oxygenation of monoterpenes (myrcene and pinenes), sesquiterpenes (farnesene), and diterpenes (abietadienol and abietic acid) (Ro et al., 2005; Zulak and Bohlmann, 2010; Hamberger et al., 2011), was only identified in dicots in a previous study (Wei and Chen, 2018). In this study, the orthologous genes of *CYP720* in orchids were identified (Supplementary Figure S12, red). Differences among CYP85 clans may involve the dendrobine signaling pathway in *D. nobile*.

Furthermore, other differences in orchid-specific related traits in CYP450 genes were also identified. CYP78 is involved in regulating organ size and cell proliferation (Nagasawa et al., 2013; Wang X. et al., 2015). More genes expanded by tandem duplication were found in *Dendrobium* species (Supplementary Figure S12, pink), such as *Dnobile03G00500.1*, *Dnobile03G00501.1*, and *Dnobile03G00502.1* in *D. nobile*, *GuChui-Maker69281*, *GuChui-Maker69269*, *GuChui-Maker69278*, *GuChui-Maker23937*, and *GuChui-Maker23933* in *D. chrysotoxum*, which may be involved in the formation of large capsules and swollen stems. CYP715, as a single gene family, plays a role as a key regulator in flower maturation, synchronizing petal expansion, and volatile emission in *Arabidopsis* (Liu et al., 2015). Interestingly, there were two to three CYP715 homologous genes in three *Dendrobium* species and *P. equestris* (Supplementary Figure S12, blue), indicating that gene expansion occurred in the Epidendroideae subfamily, potentially as a result of floral development.

## Discussion

To date, three *Dendrobium* species recorded in the Chinese Pharmacopoeia (2020 Edition) have had their complete genome sequenced. The chromosome-level genome assembly of *D. nobile* should be helpful for further functional research as the model of dendrobine biosynthesis in *Dendrobium*. As one of the most important medicinal plants in traditional Chinese medicine, *D. nobile* is mainly composed of dendrobine and polysaccharides with pharmacological activity. This study contributed to improving the understanding of the metabolism of these medicinal components.

The heterozygosity of the *D. nobile* genome is 1.35%, meaning that it is difficult to assemble (Xin et al., 2019). In this study, Pacbio Sequel II sequencing technology with longer read lengths was used. Indeed, the long sequencing reads led to a BUSCO completeness estimate of 96.22%, which was a huge improvement. Polyploidization occurs frequently in angiosperms, which contributes to plant adaption to the environment and plant genome evolution (Van de Peer et al., 2017). One polyploidization event occurred in the most recent common ancestor of orchids (Cai et al., 2015; Zhang et al., 2016; Zhang et al., 2017). In this study, *D. nobile* was estimated to have undergone a WGD event at the same time, which further confirmed that the WGD event occurred at the most recent common ancestor of orchids. Furthermore, the average length of genes and introns in orchids, with the exception of *A. shenzhenica*, was much higher than that in most other angiosperms (Zhang et al., 2017). There are regulatory elements that are frequently contained within introns, and alternative splicing events often occur among different introns and exons, diversifying the protein coding of the genome. All of these factors may contribute to the genome structure evolution, genome size, gene function diversification, and gene expression pattern of a species (Castillo-Davis et al., 2002; De La Torre et al., 2014; Sena et al., 2014; Keane and Seoighe, 2016). For example, the long intron transcriptional delay in *Drosophila* is particularly important for the proper development of the embryo (Swinburne and Silver, 2008; Artieri and Fraser, 2014). This characteristic of orchids requires further analysis and research.

The content and composition of alkaloids and polysaccharides vary with factors including the tissues sampled, species, and genetics. For the genes in the polysaccharide biosynthesis pathway, *NI* genes are localized in the mitochondria, chloroplasts, and cytosol (Roitsch and Gonzalez, 2004). The diversity of subcellular localization suggests that NIs have a variety of physiological functions. Sucrose unloaded in the sink cell can be cleaved in the cytosol by NIs, which are involved in cellulose biosynthesis (Roitsch and Gonzalez, 2004; Rende et al., 2017), suggesting that the cytosolic NIs contribute to the supply of substrate for cellulose biosynthesis (Rende et al., 2017). In this study, the number of cytosolic NIs was found to be three, four, and seven in *D. catenatum*, *D. nobile*, and *D. chrysotoxum*, respectively. These large differences in quantity may contribute to the differences in the biosynthesis of polysaccharides among *Dendrobium* species. Cytosolic phosphoglucomutase (cPGM) interconverts glucose-6-phosphate and glucose-1-phosphate and is a key enzyme in central metabolism (Egli et al., 2010). Interestingly, five cPGM members were detected in *D. chrysotoxum*, one in *D. nobile*, and one in *D. catenatum*, suggesting that different molecular mechanisms may occur among *Dendrobium* species. Sucrose-phosphate synthase (SPS; E.C. 2.4.1.14) is a plant enzyme that plays vital roles in sucrose production across various plant species and is involved in photosynthesis (Huber and Huber, 1996; Ma et al., 2020). The *SPS* genes were only found in *Dendrobium* in this study, indicating a *Dendrobium-*specific sucrose biosynthesis mechanism.

Compared with multi-gene families in the polysaccharide pathway, most of the gene families in the alkaloid pathway were single-copy, except for *DHS*, *DXS*, and *HMGR* gene families. Therefore, the main difference in genes in the alkaloid pathway among *Dendrobium* species was the gene expression pattern in stems and leaves, with the exception of the loss of several genes. The expression pattern of the upstream genes in the alkaloid biosynthesis pathway may be the main factor contributing to the differences in the content and composition of alkaloids among *Dendrobium* species.

TPSs are a diverse class of enzymes that catalyze the biosynthesis of all kinds of terpenes (McGarvey and Croteau, 1995). TPSs may have originated from isoprenyl diphosphate synthase genes, which are involved in dendrobine biosynthesis (Jiang et al., 2019; Wang et al., 2020). The *CrGES* (encoding geraniol synthase in *C. roseus*, the homologue of *TPS02*) and *TPS21* genes are involved in dendrobine biosynthesis (Simkin et al., 2013; Kuang et al., 2016; Wang et al., 2020). In this study, the number of *TPS* genes in *Dendrobium* was much higher than that in *A. shenzhenica* and *P. equestris*, indicating a relationship between *TPS* genes and dendrobine biosynthesis. Interestingly, the phylogenetic analysis revealed that there were 21, 16, and 17 TPS-a genes in *D. nobile*, *D. chrysotoxum*, and *D. catenatum*, respectively. As the TPS-a subfamily encodes only sesqui-TPSs (Jiang et al*.*, 2019), the higher number of TPS-a genes in *D. nobile* and their expression mainly in the stem may be important factors involved in the production of more dendrobine compared to other *Dendrobium* species (Li et al*.*, 2019). Furthermore, the opposite gene distribution in TPS-b-II and TPS-b-III among the three *Dendrobium* species may contribute to the differences in the amounts and constitution of alkaloids between *D. nobile* and *D. chrysotoxum* in various tissues, as the TPS-b subfamily encodes monoTPSs (Jiang et al., 2019).

For the CYP450 gene family, the main difference was found in the CYP85 clan, which was mainly involved in the species-specific synthesis and catabolism pathway of terpenoids (Schuler, 2015). There were twice as many CYP87B-C genes in *D. nobile* compared to other *Dendrobium* species. A previous study found that the CYP720 subfamily only occurred in dicots (Wei and Chen, 2018), mediating oxygenation of monoterpenes, sesquiterpenes, and diterpenes (Ro et al*.*, 2005; Zulak and Bohlmann, 2010; Hamberger et al., 2011). This subfamily was also identified in this study, suggesting a contribution to the biosynthesis of orchid-specific components. All of the differences in CYP85 clans may be involved in the dendrobine signaling pathway in *D. nobile*.

## Conclusion

Although *D. nobile* has high medicinal and ornamental value, the lack of omics data has hampered molecular mechanism research and the development of medicinal ingredients in this species. In this study, a chromosome-level reference genome of *D. nobile* was obtained, with an assembled genome size of 1.19 Gb and 29,476 annotated protein-coding genes. Two polyploidization events occurred in *D. nobile* based on *Ks* analysis: the recent WGD shared with other orchid species and the ancient polyploidization event shared with most monocots (the tau event). Phylogenetic analysis of the *D. nobile* gene family involved in the polysaccharide synthesis pathway showed that the gene number variation and *Dendrobium*-specific genes may be related to the fleshy stems with abundant polysaccharides. The analysis results of the *TPS* and *CYP450* gene families suggest that there are more TPS-a genes in *D. nobile*, and the opposite distribution pattern in TPS-b-II and TPS-b-III among *Dendrobium* species may contribute to the species-specific alkaloid biosynthesis pathways. The differences in CYP85 clans among *Dendrobium* species may also play important roles in alkaloid biosynthesis and floral development. The analysis of *D. nobile* revealed the mechanism through which the fleshy stem produces abundant polysaccharides and alkaloids, as well as the floral development regulation, which is critical for industrial development. This was the first study to fully analyze the characteristics of genes in the biosynthesis pathways of polysaccharides and alkaloids in *Dendrobium*. The results of this study provide a high-quality genome of *Dendrobium* and important insights into the molecular elucidation of medicinal active ingredients, molecular breeding, and orchid responses to the environment, enhancing the understanding of orchid evolution.

## Accession Codes and Genome Links

All data from this study were submitted to the NCBI database under Bioproject ID: PRJNA725550. This Whole Genome Shotgun project has been deposited at DDBJ/ENA/GenBank under the accession JAGYWB000000000. The version described in this paper is version JAGYWB010000000.

## Data Availability

The datasets presented in this study can be found in online repositories. The names of the repository/repositories and accession number(s) can be found in the article/Supplementary Material.
