# Supplementary material for: Chromosome-Scale Assembly of the Dendrobium nobile Genome Provides Insights Into the Molecular Mechanism of the Biosynthesis of the Medicinal Active Ingredient of Dendrobium
Source: Front Genet. 2022 Mar 1;13:844622. doi: 10.3389/fgene.2022.844622 (PMC8921531; doi:10.3389/fgene.2022.844622)
Supplement: Supplementary file 1 [file DataSheet3.pdf]

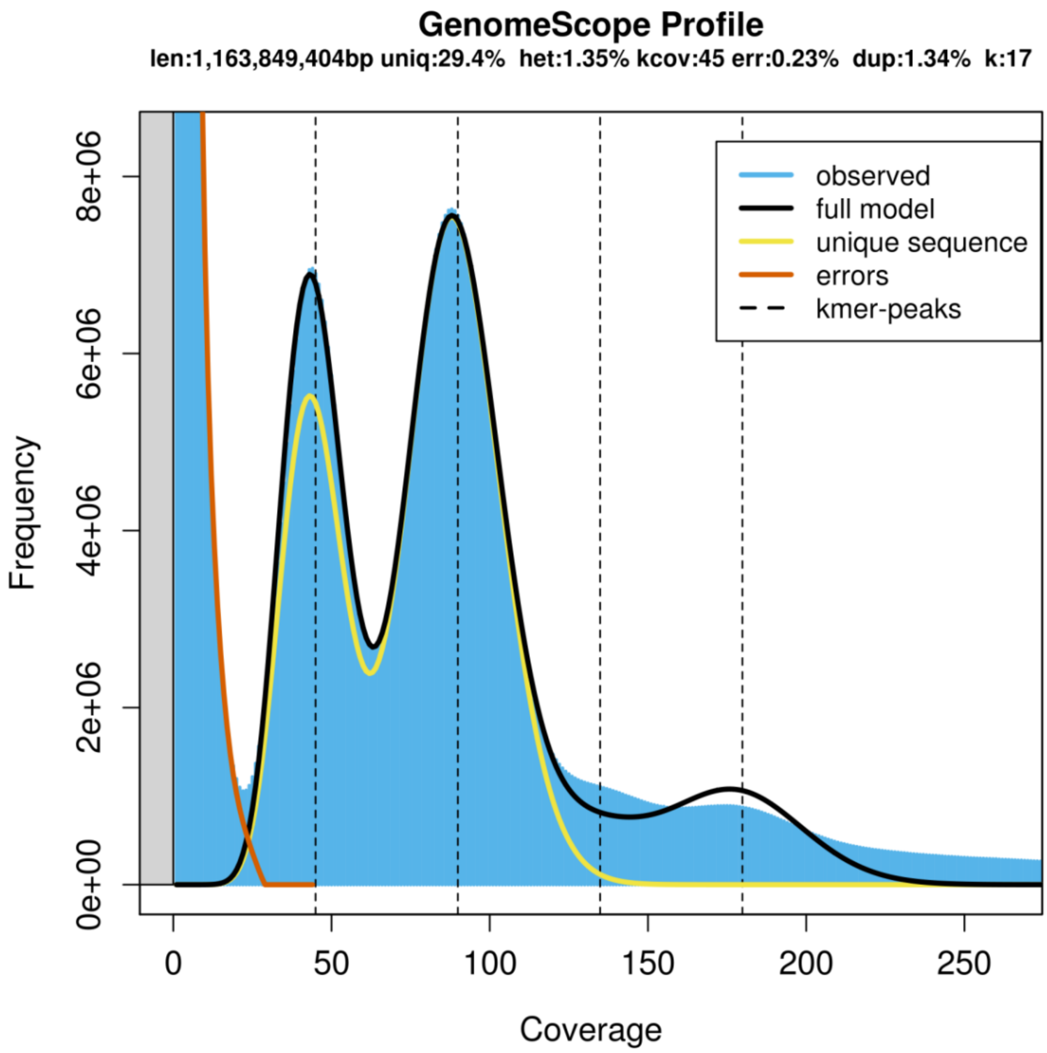

Figure S1. Genome size and heterozygosity estimation using 17 *K-mer* distribution.

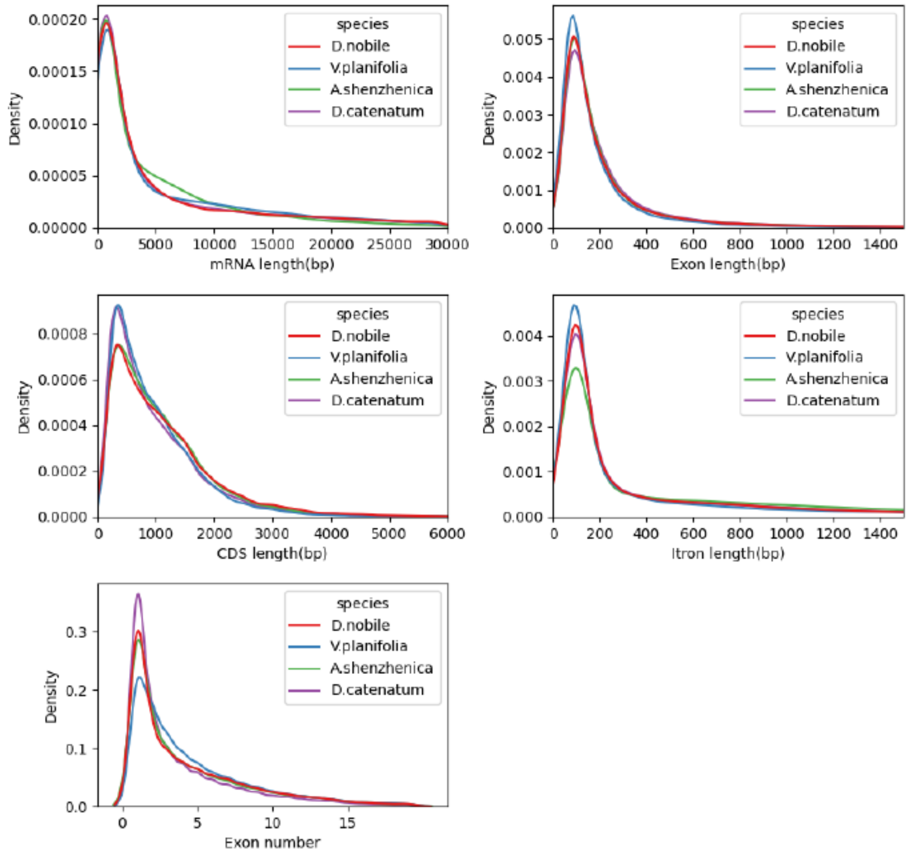

Figure S2. Gene structure prediction statistical results. *Dendrobium nobile* compared with the genetic elements of related species. Window refers to the length represented by each point on the horizonal coordinate.

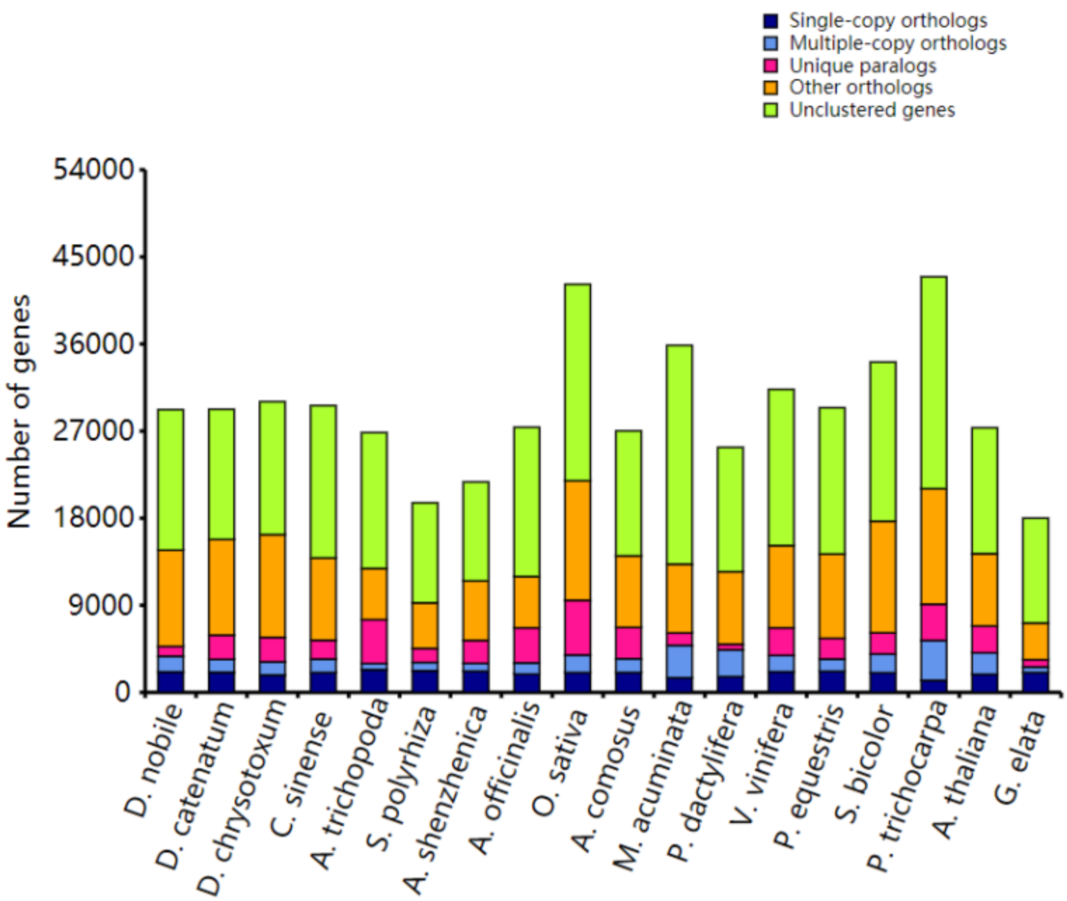

Figure S3. Classification and distribution of homologous gene families among species.

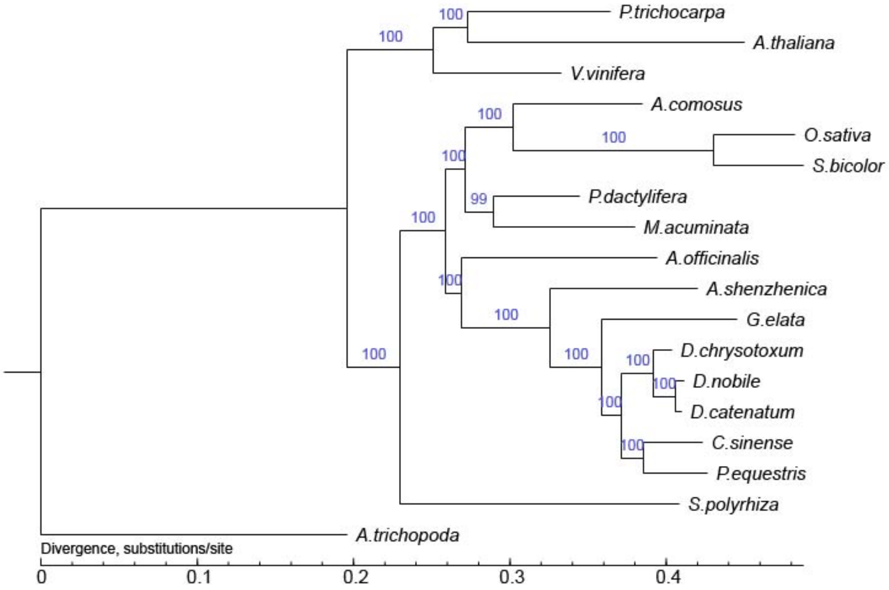

Figure S4. Phylogenetic tree constructed based on the filtered single-copy gene family; each branch length represents the neutral evolution rate.

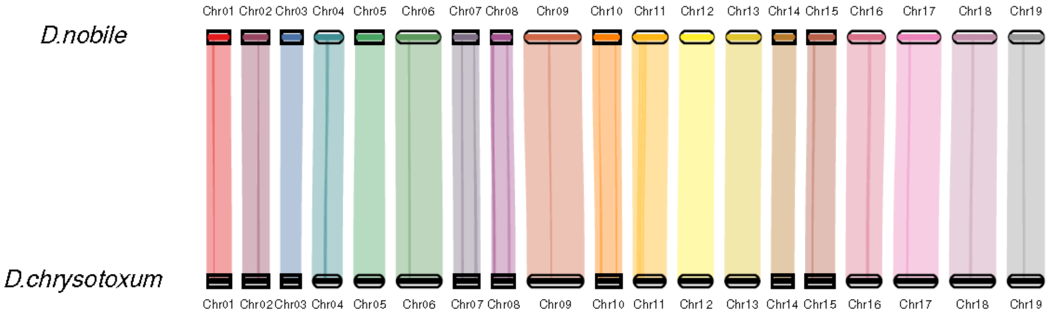

Figure S5. Collinear diagram between *Dendrobium nobile* and *Dendrobium chrysotoxum.*

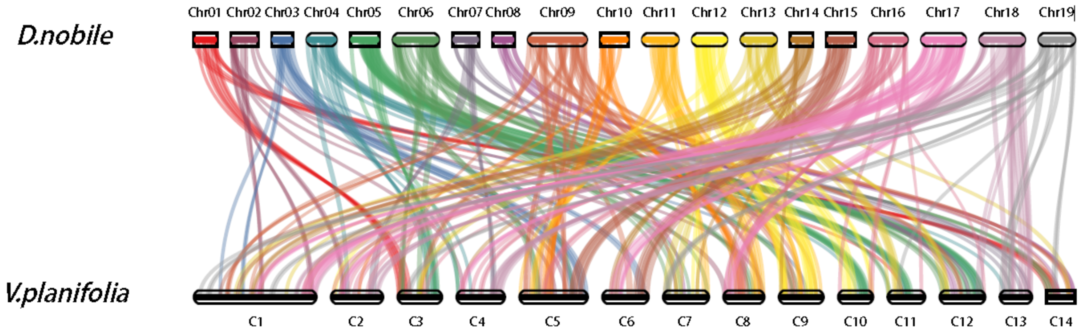

Figure S6. Collinear diagram between *Dendrobium nobile,* and *Vanilla planifolia*.

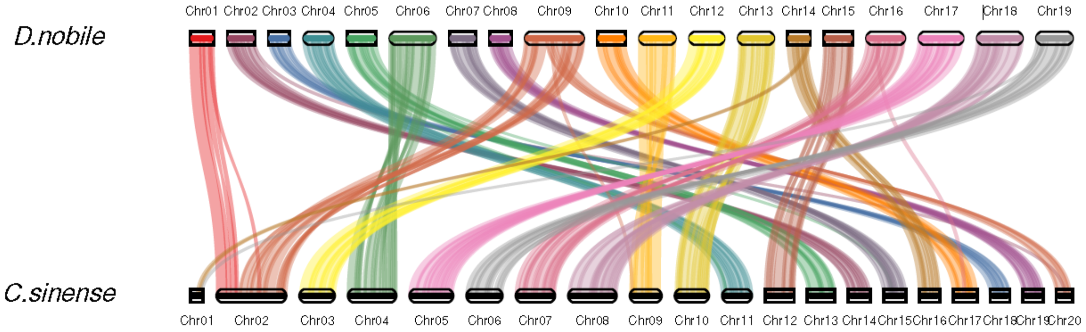

Figure S7. Collinear diagram between *Dendrobium nobile,* and *Cymbidium sinense*.

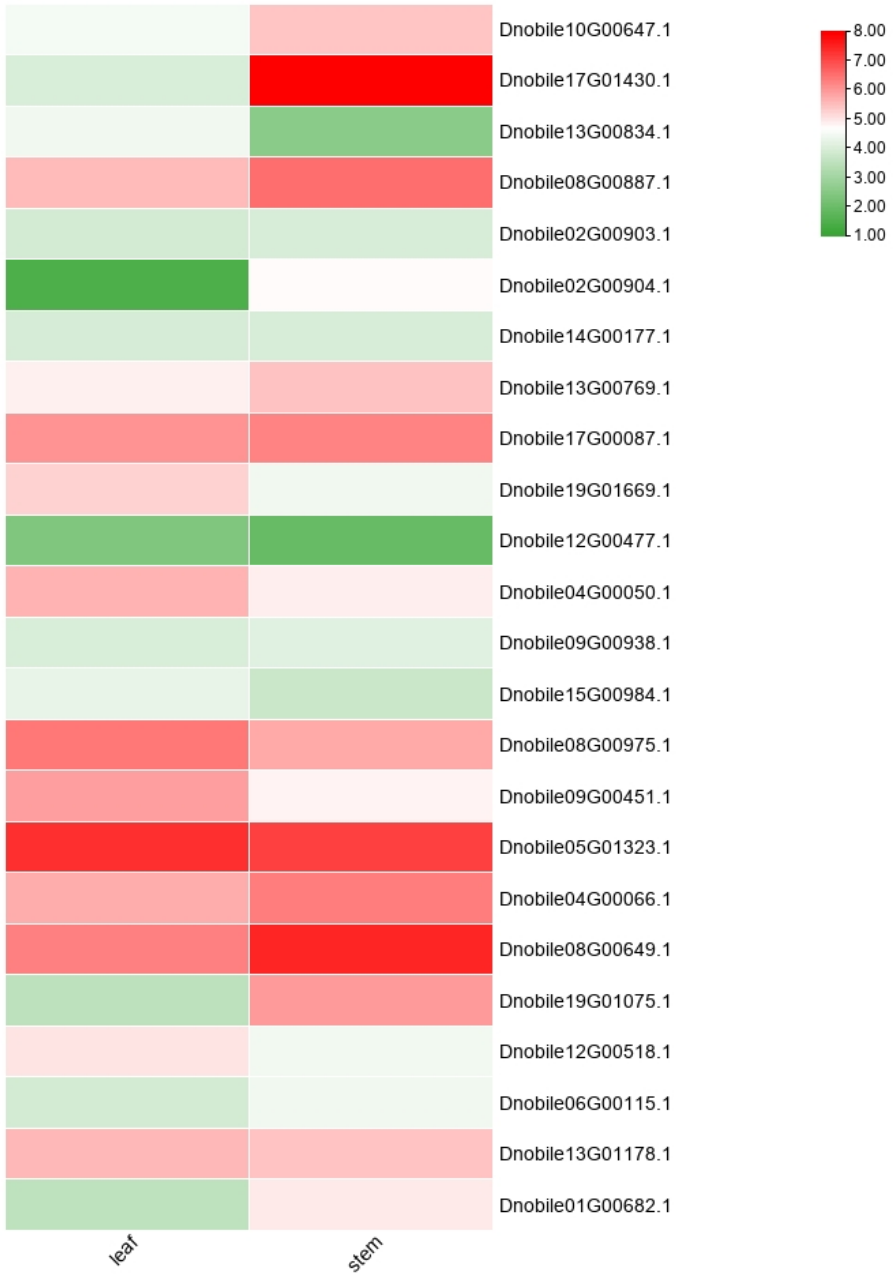

Figure S8. Expression patterns of genes upstream of the alkaloid biosynthesis pathway in the leaf and stem tissue of *Dendrobium nobile*. The expression levels (Log10 transformed of FPKM value) are represented by the color bar.

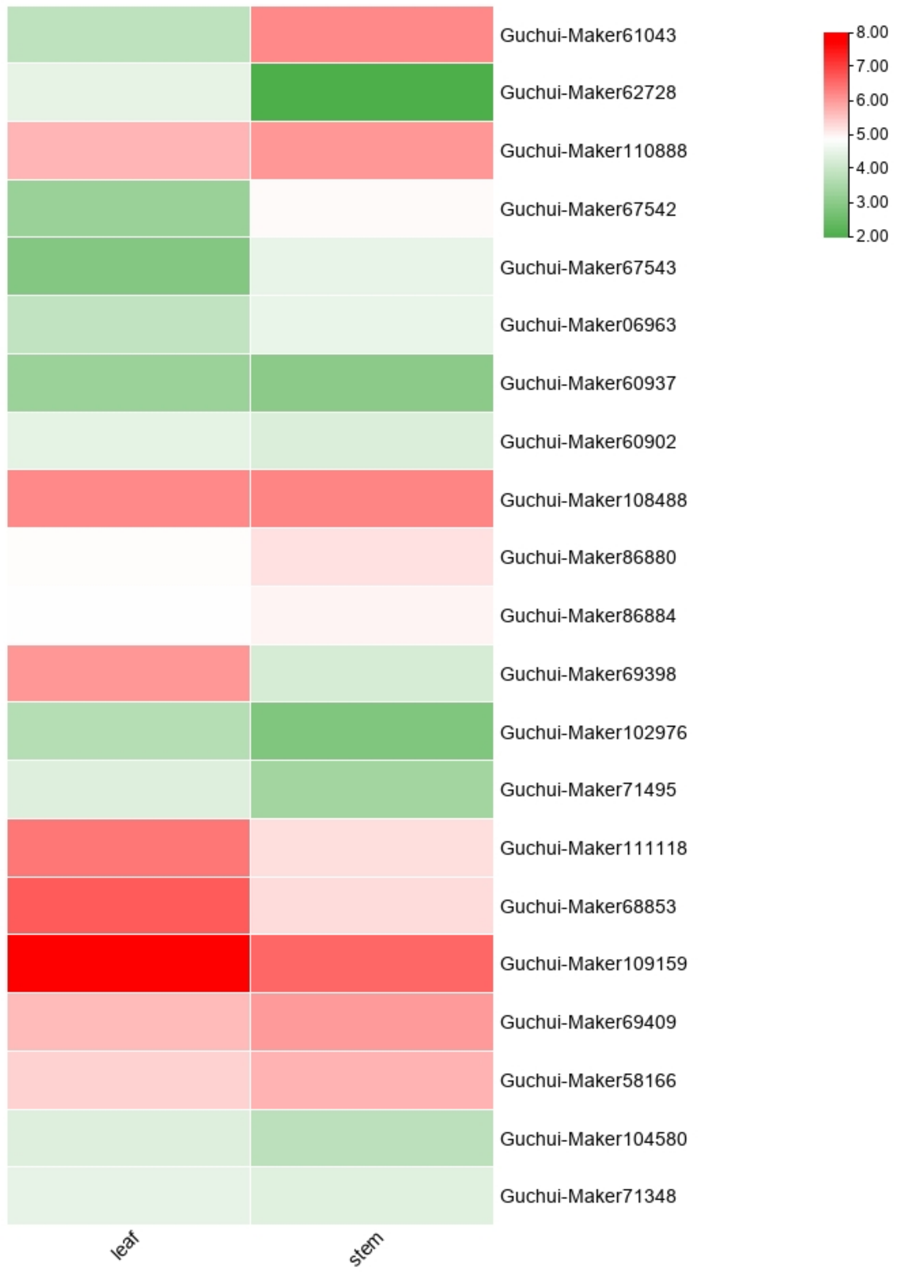

Figure S9. Expression patterns of genes upstream of the alkaloid biosynthesis pathway in the leaf and stem tissue of *Dendrobium chrysotoxum*. The expression levels (Log10 transformed of FPKM value) are represented by the color bar.

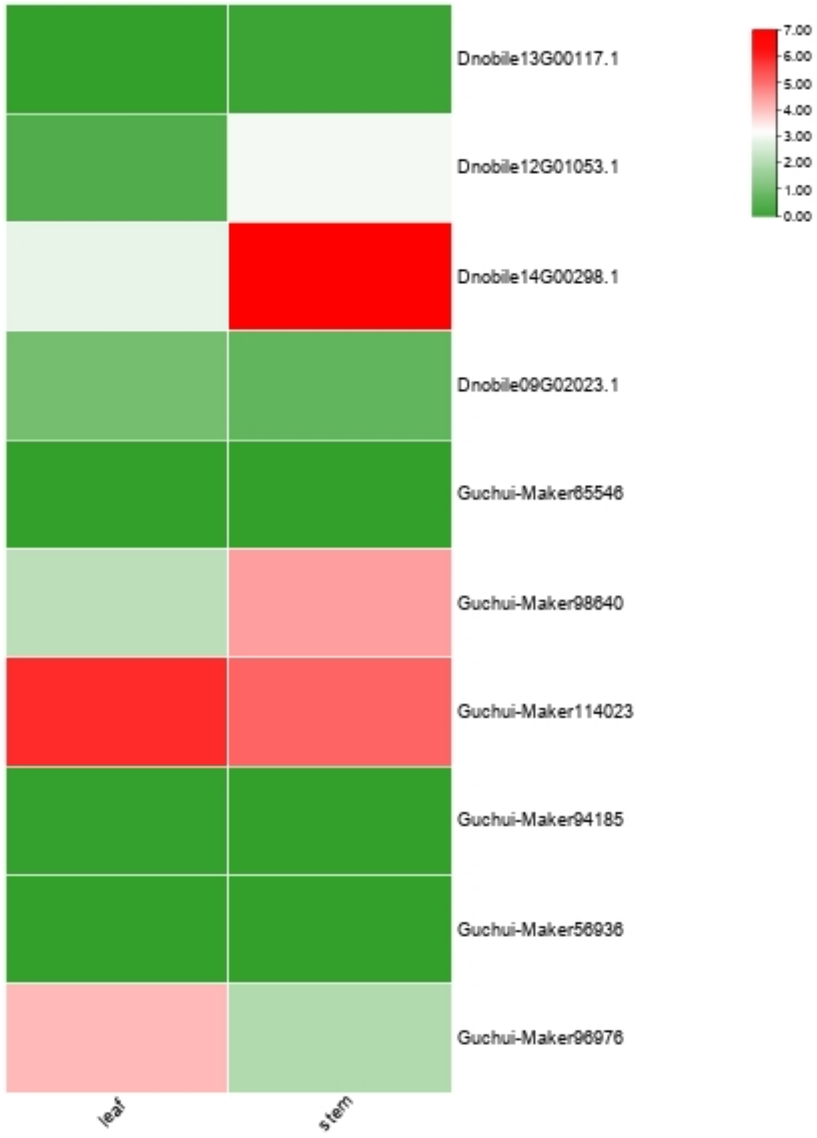

Fig S10. The *STR* gene expression pattern in the leaf and stem tissue of *Dendrobium nobile* and *Dendrobium chrysotoxum*. The expression levels (Log10 transformed of FPKM value) are represented by the color bar.

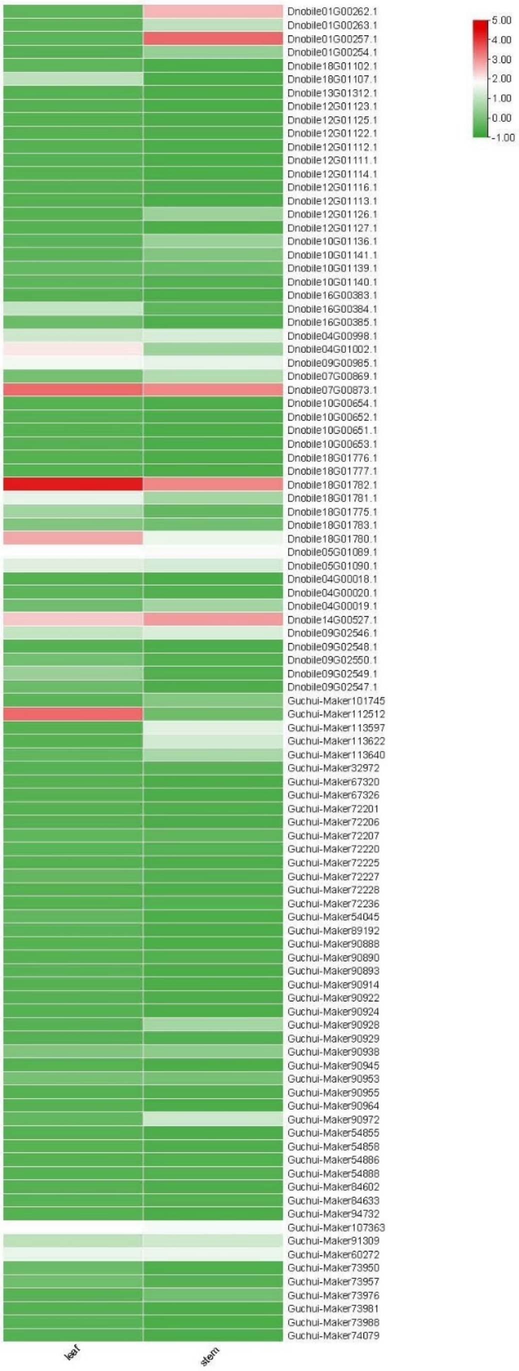

Fig S11. The *TPS* gene expression pattern in the leaf and stem tissue of *Dendrobium nobile* and *Dendrobium chrysotoxum*. The expression levels (Log10 transformed of FPKM value) are represented by the color bar.

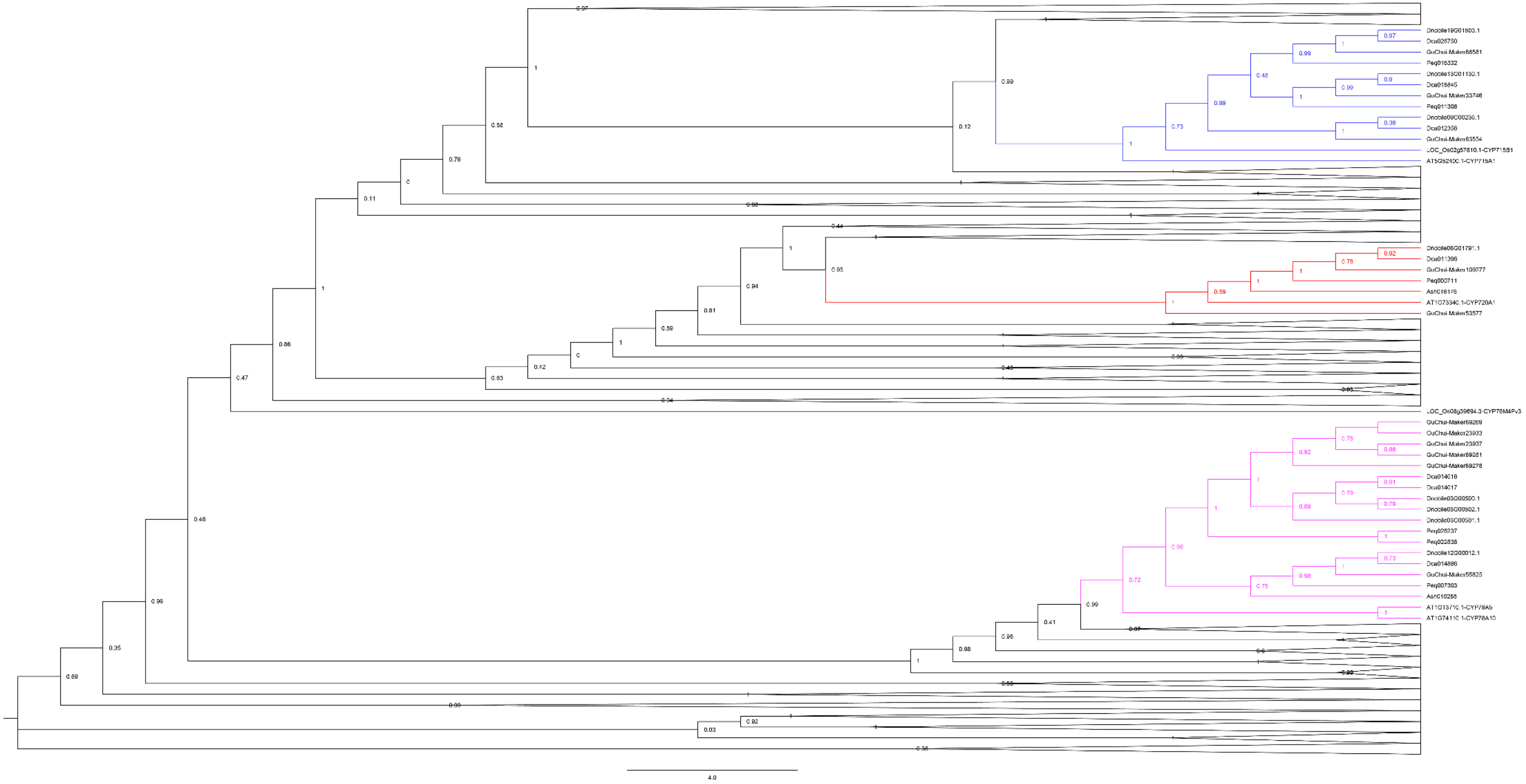

Figure S12. Phylogenetic analysis of *CYP450* genes in *Dendrobium nobile*, *Dendrobium chrysotoxum*, *Dendrobium catenatum*, *Apostasia shenzhenica*, *Phalaenopsis equestris*, *Arabidopsis thaliana*, and *Oryza sativa*. Dnobile, *D. nobile*; Guchui-Maker, *D. chrysotoxum*; Dca, *D. catenatum*; Ash, *A. shenzhenica*; Peq, *P. equestris*; AT, *A. thaliana*; LOC_Os, *Oryza sativa*. The CYP720 subfamily is shown in red. Pink indicates the CYP78 subfamily. CYP715 is shown in blue.
