# Supplementary material for: Chromosome-Scale Assembly of the Dendrobium nobile Genome Provides Insights Into the Molecular Mechanism of the Biosynthesis of the Medicinal Active Ingredient of Dendrobium
Source: Front Genet. 2022 Mar 1;13:844622. doi: 10.3389/fgene.2022.844622 (PMC8921531; doi:10.3389/fgene.2022.844622)
Supplement: Supplementary file 2 [file DataSheet1.pdf]

Table S1. Summary of sequencing data.

Sequencing Platform	Read length(bp)	Raw Base (Gb)	Sequence coverage (X)
MGISEQ-2000	150	130.62	109.8
Pacbio Sequel II	14,009.37	96.91	81.4
Hi-C	150	245.48	206.29

Table S2. Chromosome length by Hi-C assembly.

Chromosome ID	Number of contig in chromosome	Size (bp)
Chr01	68	57,995,321
Chr02	61	64,459,299
Chr03	60	39,261,714
Chr04	118	76,253,716
Chr05	93	61,811,326
Chr06	149	90,061,196
Chr07	63	53,775,998
Chr08	52	37,776,844
Chr09	104	81,924,558
Chr10	64	41,420,473
Chr11	94	58,136,154
Chr12	49	51,326,659
Chr13	85	55,952,746
Chr14	67	44,468,460
Chr15	77	46,061,428
Chr16	96	74,496,069
Chr17	80	77,942,481
Chr18	89	95,358,005
Chr19	96	84,049,299

Table S3. Summary of assembly results.

	Contig		Scaffold	
	Size(bp)	Number	Size(bp)	Number
N10	5,229,378	17	90,061,196	2
N20	3,513,425	45	84,049,299	3
N30	2,647,333	86	77,942,481	5
N40	2,049,888	137	76,253,716	6
N50	1,618,306	204	64,459,299	8
N60	1,298,484	287	58,136,154	10
N70	1,004,915	392	55,952,746	12
N80	696,039	534	51,326,659	14
N90	392,755	764	41,420,473	17
Max Length	16.050,566	--	95,358,005	--
Total number	--	1603	--	57
Total Length	1,198,962,375	--	1,199,116,975	--
GC Ratio	0.353	--	0.353	--

Table S4. BUSCO assessment of the *Dendrobium nobile* genome assembly.

Type	Number	Percentage
Complete BUSCOs（C）	1,553	96.22%
Complete and single-copy BUSCOs (S)	1,508	93.43%
Complete and duplicated BUSCOs (D)	45	2.79%
Fragmented BUSCOs (F)	24	1.49%
Missing BUSCOs (M)	37	2.29%
Total BUSCO groups searched	1,614	

Table S5. Mapping statistics of short reads.

Total reads	Mapped reads	Mapped and paired reads	Properly paired reads	Mapped rate（%）	Mapped and paired rate（%）	Properly paired rate
848862162	838883774	837894510	808285086	98.82	98.71	95.22

Table S6. Prediction of gene structures in *Dendrobium nobile*.

	Gene set	Number	Average gene length(bp)	Average CDS length(bp)	Average exon per gene	Average exon length(bp)	Average intron length(bp)
De novo	Augustus	132,301	4787.17	1184.18	3.15	375.85	1675.31
	Snap	212,076	6813.79	832.49	3.82	218.16	2124.14
Homolog	*A.shenzhenica*	26,182	7505.79	1072.6	3.9	275.15	2219.71
	A.thaliana	18,669	10589.97	1210.2	5.22	231.92	2223.6
	A.officinalis	18,296	10423.67	1191.53	5.34	223.22	2128.26
	G.elata	16,367	10456.19	1255.9	5.12	245.07	2230.5
	O.sativa	18,898	10293.38	1237.87	5.13	241.4	2193.76
	P.equestris	20,005	10397.17	1253.27	5.14	243.74	2207.7
	V.planifolia	20,821	9425.22	1127.82	4.88	231.31	2140.85
RNA-seq	transcript	56,303	18509.95	1007.86	4.63	217.6	4819.27
Maker	-	29,476	11550.65	1146.11	4.55	251.83	2930.01

Table S7. Statistical results of gene sets

	>=30% overlap		>=50% overlap		>=80% overlap	
	No.	Ratio	No.	Ratio	No.	Ratio
R	1,579	5.36	1,748	5.93	2,400	8.14
H(single)	38	0.13	51	0.17	111	0.38
H(more)	18	0.06	39	0.13	213	0.72
P(single)	1,217	4.13	1,520	5.16	2,595	8.80
P(more)	2,241	7.60	2,304	7.82	1,852	6.28
HR	152	0.52	295	1.00	1,380	4.68
PR	2,351	7.98	2,222	7.54	2,157	7.32
PH	3,151	10.69	3,288	11.15	3,343	11.34
PHR	18,301	62.09	17,502	59.38	14,519	49.26
Total	29,048	98.55	28,969	98.28	28,570	96.93

Note: R shows gene prediction supported by RNA-sequencing (RNA-seq); P suggests gene prediction by de novo methods; H means gene prediction by homolog methods; single: gene prediction by only one-time data supported; more: gene prediction by many time data supported; overlap: the ratio of the final gene set to the CDS region of each predicted result.

Table S8. BUSCO assessment of the *Dendrobium nobile* genome prediction.

Type	Number	Percentage (%)
Complete BUSCOs（C）	1,508	93.43
Complete and single-copy BUSCOs (S)	1,446	89.59
Complete and duplicated BUSCOs (D)	62	3.84
Fragmented BUSCOs (F)	58	3.59
Missing BUSCOs (M)	48	2.97
Total BUSCO groups searched	1,614	-

Table S9. Statistics of non-coding RNA in the *Dendrobium nobile* genome.

Type	Copy	Average length (bp)	Total length (bp)	% of genome
miRNA	76	127.25	9,671	0.000807
tRNA	386	75.3601	29,089	0.002426
rRNA	958	177.7902	170,323	0.014206
18S	261	385.092	100,509	0.008383
28S	119	125.1092	14,888	0.001242
5.8S	32	109.625	3,508	0.000293
5S	546	94.17216	51,418	0.004289
snRNA	457	114.5405	52,345	0.004366
CD-box	272	105.1654	28,605	0.002386
HACA-box	24	130.875	3,141	0.000262
splicing	161	127.9441	20,599	0.001718

Table S10. Statistical results of repeat sequences.

Type	Repeat Size(bp)	% of genome
TRF	47,646,963	3.974017
RepeatMasker	164,446,337	13.715721
RepeatProteinMask	172,675,014	14.402038
De novo	695,501,841	58.008646
Total	732,188,741	61.068534

Table S11. Statistics of repeat sequences in *Dendrobium nobile*.

	Type	Length(bp)	% of genome
Retro	LTR/Copia	349,879,529	29.18186
	LTR/Gypsy	253,972,999	21.18273
	LTR/Other	11,376,510	0.94886
	SINE	13,596	0.00113
	LINE	61,853,346	5.15891
	Other	43,021	0.00359
DNA	EnSpm	7,918,050	0.66041
	Harbinger	645,889	0.05387
	hAT	10,062,319	0.83925
	Helitron	1,588,457	0.13249
	Mariner	92	1.00E-05
	MuDR	2,787,978	0.23253
	P	0	0
	Other	700,820	0.05845
Other	-	1,020,027	0.08508
Unknown	-	24,129,963	2.01257
Total	-	695,501,841	58.00865

LINE, long interspersed nuclear element; SINE, short interspersed element; LTR, long terminal repeat.
Unknown repeat sequences could not be clustered by Repeat Masker.

Table S12. Statistical results of functional annotation.

Values	Total	Nr	Swissprot	KEGG	KOG	TrEMBL	Interpro	GO	Overall
Number	29,476	27,601	19,855	20,215	19,801	25,870	24,044	3,583	27,765
Percentage	-	93.64%	67.36%	68.58%	67.18%	87.77%	81.57%	12.16%	94.20%

Table S13. The statistics of gene families clustering in *Dendrobium nobile*.

Species	Genes number	Genes in families	Unclustered genes	Family number	Unique families	Average genes per family
D.nobile	**29214**	**23220**	**5994**	**14860**	**361**	**1.56**
D.catenatum	29257	23663	5594	15338	825	1.54
D.chrysotoxum	30045	24209	5836	14012	552	1.73
C.sinense	29638	21776	7862	14202	716	1.53
A.trichopoda	26846	19399	7447	12493	1048	1.55
S.polyrhiza	19592	16299	3293	11547	363	1.41
A.shenzhenica	21743	18622	3121	11994	487	1.55
A.officinalis	27395	19848	7547	12113	863	1.64
O.sativa	42173	31032	11141	17136	1335	1.81
A.comosus	27024	22201	4823	13128	692	1.69
M.acuminata	35864	25477	10387	12697	518	2.01
P.dactylifera	25321	22834	2487	12730	219	1.79
V.vinifera	31315	23950	7365	14070	924	1.7
P.equestris	29413	22096	7317	14185	570	1.56
S.bicolor	34129	26911	7218	16439	764	1.64
P.trichocarpa	42945	33831	9114	14533	1154	2.33
A.thaliana	27342	23587	3755	12844	716	1.84
G.elata	18000	13895	4105	10828	264	1.28

Table S14. KEGG enrichment results of significantly expanded gene families in *Dendrobium nobile* (see separate files).

Table S15. Statistical results of whole gene collinearity analysis.

Species1	Species2	Number of Synteny Blocks	Number of Synteny Genes in All Blocks
D. nobile	*D. chrysotoxum*	120	31,498
D. nobile	*V. planifolia*	1,404	25,166
D. nobile	*C. sinense*	430	30,650

Table S16. The genes numbers in the polysaccharide synthesis pathway in five orchid species and *Arabidopsis thaliana*.

Gene family		Ash	Peq	Dca	Guchui	JC	AT
NI	Cytosolic	3	3	3	7	4	5
	Mitochondria	2	2	3	1	2	3
	Chloroplastic	1	1	1	1	1	1
PGM	pPGM	1	1	1	1	1	1
	cPGM	1	1	1	5	1	2
SPS	SPS1/2F	1	2	1	1	1	2
	SPS3F	1	1	1	1	1	1
	SPS4F	0	0	1	1	1	1
SUS	SUS1/4	2	3	3	2	2	2
	SUS2/3	1	1	1	1	1	2
	SUS5/6	1	2	1	1	1	2
UGP	UGP1/2	1	1	1	1	1	2
	UGP3	1	1	1	1	1	1
UGE	GROUP1	3	5	4	6	**5**	5
	GROUP2	5	4	5	4	**0**	6

Species abbreviations are as follows: Ash, *Apostasia shenzhenica*; Peq, *Phalaenopsis equestris*; Dca, *Dendrobium catenatum*; Guichui, *Dendrobium chrysotoxum*; JC, *Dendrobium nobile*; AT, *Arabidopsis thaliana*.
Genes encoding enzymes abbreviations are as follows: PGM, phosphoglucomutase; UGP, UDP-glucose pyrophosphorylase; SPS, sucrose-phosphate synthase; SUS, sucrose synthase; UGE, UDP galacturonate 4-epimerase; NI, Alkaline/neutral invertase.
Green color shows the varying gene numbers in different gene family branches; yellow color shows the *Dendrobium*-specific genes.

Table S17. The gene numbers in the upstream of the alkaloid biosynthesis pathway.

Gene family		Ash	Peq	Dca	Guchui	JC	AT
Shikimate pathway	DHS	2	2	3	2	3	3
	DHQS	1	1	2	1	1	1
	DHD-SKDH	1	1	1	1	2	1
	SK	1	1	1	1	1	2
	SHKG	1	1	1	2	1	2
	CS	1	1	1	1	1	1
MEP pathway	DXS	2	5	2	2	2	2
	DXR	1	1	1	1	1	1
	CMS	1	1	1	1	1	1
	CMK	1	1	1	1	1	1
	MCS	1	1	1	1	1	1
	HDS	1	1	1	1	1	1
	HDR	1	1	1	1	1	1
MVA pathway	AACT	2	1	1	1	1	2
	HMGS	1	1	2	1	1	1
	HMGR	3	2	4	0	2	2
	MVK	1	1	1	1	1	1
	PMK	1	1	1	1	1	1
	MVD	1	1	1	0	1	2
STR	STR5/6/7/8	0	0	0	0	0	4
	STR10	0	1	2	1	1	1
	STR12/13	1	0	1	2	1	2
	STR11	1	0	1	1	1	1
	STR9	2	2	1	2	1	1
